# Single-cell quantification of ribosome occupancy in early mouse development

**DOI:** 10.1038/s41586-023-06228-9

**Published:** 2023-06-21

**Authors:** Hakan Ozadam, Tori Tonn, Crystal M. Han, Alia Segura, Ian Hoskins, Shilpa Rao, Vighnesh Ghatpande, Duc Tran, David Catoe, Marc Salit, Can Cenik

**Affiliations:** 1 Department of Molecular Biosciences, University of Texas at Austin, Austin, TX USA; 2 Department of Mechanical Engineering, San Jose State University, San Jose, CA USA; 3 Department of Chemical and Materials Engineering, San Jose State University, San Jose, CA USA; 4 Joint Initiative for Metrology in Biology, SLAC National Accelerator Laboratory, Menlo Park, CA USA; 5 Department of Bioengineering, Stanford University, Stanford, CA USA; 6 Department of Pathology, Stanford University, Stanford, CA USA

**Keywords:** Gene expression analysis, Lab-on-a-chip, Embryogenesis, Translation

## Abstract

Translation regulation is critical for early mammalian embryonic development^1^. However, previous studies had been restricted to bulk measurements^2^, precluding precise determination of translation regulation including allele-specific analyses. Here, to address this challenge, we developed a novel microfluidic isotachophoresis (ITP) approach, named RIBOsome profiling via ITP (Ribo-ITP), and characterized translation in single oocytes and embryos during early mouse development. We identified differential translation efficiency as a key mechanism regulating genes involved in centrosome organization and *N*^6^-methyladenosine modification of RNAs. Our high-coverage measurements enabled, to our knowledge, the first analysis of allele-specific ribosome engagement in early development. These led to the discovery of stage-specific differential engagement of zygotic RNAs with ribosomes and reduced translation efficiency of transcripts exhibiting allele-biased expression. By integrating our measurements with proteomics data, we discovered that ribosome occupancy in germinal vesicle-stage oocytes is the predominant determinant of protein abundance in the zygote. The Ribo-ITP approach will enable numerous applications by providing high-coverage and high-resolution ribosome occupancy measurements from ultra-low input samples including single cells.

## Abstract (web summary)

A single-cell ribosome profiling method can provide data at the level of allele-specific ribosome engagement in early development.

## Main

The early gene expression landscape is shaped by post-transcriptional regulation of maternal transcripts due to the absence of transcription from later stages of oocyte maturation through the early divisions of the embryo^3,4^. Consequently, RNA expression and protein abundance are only modestly correlated until the late morula stage, emphasizing the need to elucidate post-transcriptional regulation during initial stages of embryogenesis^1,5,6^. In particular, translational control of specific transcripts is essential for oocyte maturation and the oocyte-to-embryo transition^7,8^.

Transcriptome-wide mRNA translation can be measured by high-throughput sequencing of RNA fragments protected by ribosomes from nuclease digestion^9,10^. However, the conventional ribosome profiling approach involves multiple steps with substantial loss of input material, restricting its application to samples with large numbers of cells. Consequently, many important questions related to translational control remain to be addressed owing to limited availability of biological material.

To overcome this constraint, we developed a method leveraging the principles of microfluidic on-chip ITP for isolation of ribosome protected fragments (RPFs). ITP has previously been applied for extraction of nucleic acids from blood, urine and cell culture samples^11^. Compared with conventional RNA extraction approaches, ITP offers faster processing times, no requirement of liquid transfers and high yield with low RNA inputs^12,13^. Despite these advantages, ITP is considered to lack the ability to deliver the stringent size selection that would be required for applications such as ribosome profiling^14,15^.

## Ribo-ITP

**Fig. 1:**
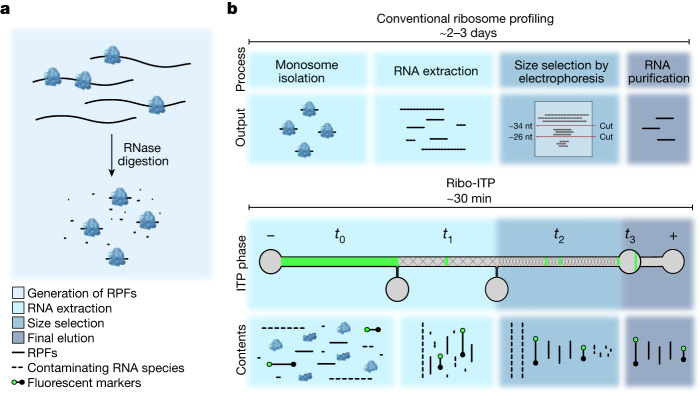
Schematic of Ribo-ITP. **a**, Schematic of the generation of RPFs. Following RNase digestion, RPFs are isolated with the conventional or Ribo-ITP approach. **b**, Schematic of the conventional ribosome profiling protocol and the Ribo-ITP process for extraction of RPFs. In Ribo-ITP, marker oligonucleotides with a 5′ fluorophore (green circle) and 3′ ddC blocking modification (black circle), which encapsulate the size range of RPFs, are added to the digested cellular lysate. Lysate contents are loaded into the channel (*t*_0_), then an electrical current is applied to a selectively focus species of a specific electrophoretic mobility range, enabling nucleic acid extraction by ITP. Nucleic acids are extracted in a narrow ITP band and then size selected as they migrate through 5% (*t*_1_) and 10% (*t*_2_) polyacrylamide gels, respectively. At the end of the run, purified and size-selected RNAs are collected (*t*_3_).

**Extended Data Fig. 1:**
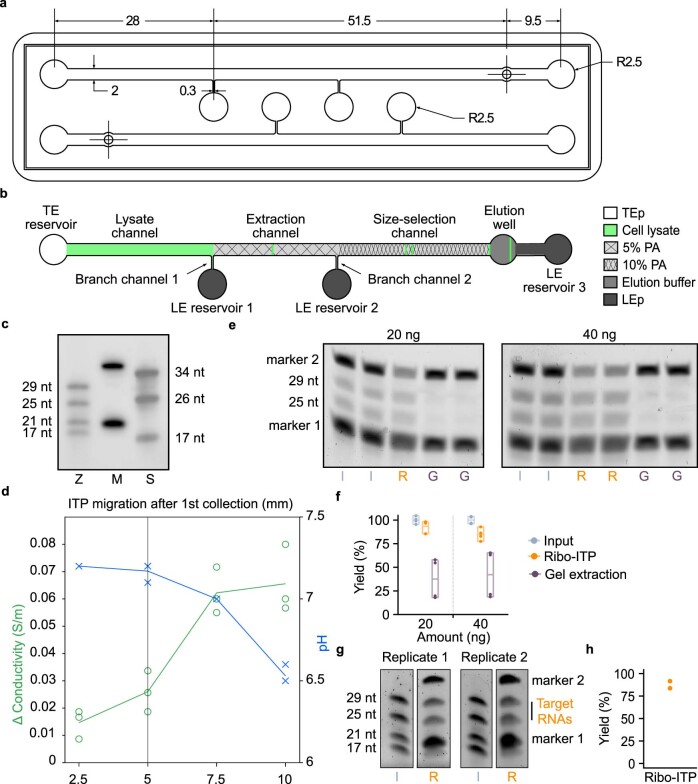
Ribo-ITP design enables efficient RNA extraction and size selection. **a**, The top view of the ITP chip layout designed with SOLIDWORKS (units in mm). The thickness of the channel features was 375 µm and that of the rectangular base was 1.5 mm. Linear tapering was applied from the rectangular base to the outer edge (rounded rectangle). **b**, Microfluidic device setup; indicating lysate, extraction, and size-selection channels; trailing electrolyte (TE) and leading electrolyte (LE) reservoirs; and elution well. Buffers corresponding to each channel and reservoir are color coded. Marker oligonucleotide fluorescence is denoted by green. **c**, Gel image displays the relative mobilities of fluorescent markers used in Ribo-ITP (M) along with the Zymo Research R1090 small RNA ladder (Z) and synthetic RNA oligonucleotides (S) (n = 1). **d**, Given that pH is the critical factor determining dephosphorylation efficiency, we determined the impact of Ribo-ITP collection on the conductivity and pH of the dephosphorylation buffer. We found negligible pH change (right axis, blue) and only a 11.0% ± 1.83% (SEM) change in conductivity (left axis, green) for the collection distance (5mm, denoted by the vertical line) used in Ribo-ITP. **e**, Representative gel images of control inputs (I), Ribo-ITP elutions (R), and gel extraction (G) samples. Four RNA species (17, 21, 25, and 29 nt) were used with total inputs of 20 and 40 ng. Fluorescent marker oligonucleotides were spiked into control and gel extraction samples prior to gel visualization. **f**, Gel image quantification of control inputs (gray), Ribo-ITP elutions (orange), and gel extraction (purple) samples (n = 4). Minimum, maximum, and average values are represented by the box and the horizontal bar. Only the 25 and 29 nt RNA marker bands were quantified for the yield calculation. **g**, Inputs (I, gray) were prepared by adding 40 ng of RNA to lysates from ~1,000 K562 cells. The RNA consisted of four species ranging from 17 to 29 nt in length. Fluorescent marker DNAs were added to Ribo-ITP samples (R) in addition to EGTA (10 mM). RNA extraction and isolation was done with Ribo-ITP followed by visualization using gel electrophoresis. **h**, Yield of the 25 and 29 nt RNAs was quantified and plotted for two replicates (84% and 91%).

Here we designed and manufactured a custom microfluidic polydimethylsiloxane (PDMS) chip to recover ribosome footprints from nuclease-digested lysates with high yield using a specialized technique named Ribo-ITP (Fig. 1a,b, Extended Data Fig. 1a,b and Supplementary Video 1). We implemented numerous innovations that enable a chemistry required to achieve single-cell ribosome profiling by coupling ITP with an optimized on-chip size selection. Specifically, we leveraged pretreatment of the channel with benzophenone to enable light-induced polymerization of polyacrylamide inside PDMS chips^16^. To aid visualization, we included DNA oligonucleotide markers containing a 5′ fluorophore and 3′ dideoxycytosine (ddC) modification to prevent marker amplification in downstream library preparation (Extended Data Fig. 1c). An on-chip buffer exchange allowed the purified RNAs to be directly compatible with 3′ dephosphorylation, the first step in sequencing library preparation of RPFs (Extended Data Fig. 1d). Finally, we adopted an efficient single-tube library preparation chemistry that relies on a template switching reverse transcriptase and incorporation of unique molecular indexes at the 5′ end of the RPFs. Collectively, Ribo-ITP reduces sample requirements by many orders of magnitude while simultaneously reducing sample processing time to deliver ribosome occupancy measurements from ultra-low input samples, including single cells. A detailed protocol including video instructions of the described Ribo-ITP method may be accessed at https://ceniklab.github.io/ribo_itp.

## Ribo-ITP RNA extraction and size selection

**Fig. 2:**
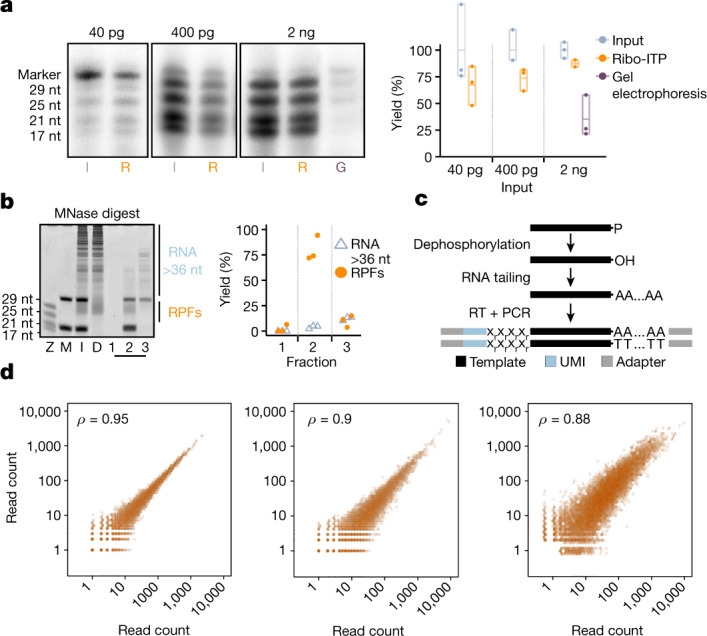
Characterization of the Ribo-ITP method and validation of efficacy in ultra-low input ribosome profiling. **a**, Representative gel images highlighting inputs (I), RNAs recovered by Ribo-ITP (R) and gel electrophoresis (G) are shown (left). Four RNAs of 17, 21, 25 and 29 nt (Z) used in the experiment were radioactively labelled at their 5′ end. The per cent yield was calculated for the 25 nt RNA (right). **b**, Representative gel image of a size selection experiment (*n* = 3 from two independent experiments). Of MNase-digested RNA from K562 cells (D), 100 ng was used as an input (I) for Ribo-ITP after the addition of the two fluorescent marker oligonucleotides (M). In a typical experiment, we collected the sample flanked by the two fluorescent nucleotide markers (fraction 2). Here we also collected the RNAs that eluted before the arrival of the shorter fluorescent marker (fraction 1) as well as the RNAs that were located behind the longer fluorescent marker (fraction 3), which typically remain in the channel. The per cent yield of RNAs larger than the longer fluorescent marker oligonucleotide (more than approximately 36 nt) (blue) and RNAs flanked by the markers (orange), corresponding to the size range of RPFs, are plotted for each fraction. **c**, Schematic of the sequencing library preparation protocol. In a single-tube reaction, isolated RPFs are 3′ dephosphorylated and poly(A)-tailed. A template-switching reverse transcriptase (RT) creates templates that incorporate unique molecular index-containing adapters. **d**, Pairwise correlation of gene-level ribosome occupancy measured in conventional ribosome profiling and Ribo-ITP from human K562 cells (right plot). The left plot highlights two replicates of conventional ribosome profiling experiments from approximately 10 million cells. The middle plot is from two replicates of Ribo-ITP with approximately 100 cells. For the right plot, we used the mean number of counts per million reads for each gene. The Spearman correlation coefficients between the gene-level ribosome occupancies are indicated in the top left corner.

Given that a typical mammalian cell contains approximately 10–40 pg of RNA, an approach capable of generating ribosome occupancy measurements from such limiting amounts needs to maintain consistently high yield of RPF recovery with inputs in the picogram range. We first compared the recovery of RNAs that span the typical size range of RPFs (approximately 21–35 nucleotides (nt)) achieved by the conventional gel extraction-based method versus Ribo-ITP. When using 20 ng input samples, Ribo-ITP yielded 94 ± 3.5% (s.e.m.) recovery in contrast to only 38 ± 10.9% for the conventional gel extraction approach (Extended Data Fig. 1e,f). We then adopted a radioactive labelling assay to visualize and quantify the recoveries from ultra-low RNA inputs (40 pg to 2 ng). With a 2 ng RNA input, 87.5 ± 3.2% yield was achieved by Ribo-ITP compared with 35.3 ± 11.4% by conventional gel extraction (Fig. 2a). When RNA inputs were decreased further to 400 pg and 40 pg, the recovery by Ribo-ITP remained high at 74 ± 6.1% and 67.5 ± 10.6%, respectively (Fig. 2a). Gel extraction had negligible yield with these samples. Thus, the consistently high RNA yields obtained with Ribo-ITP demonstrate that this method empowers high-yield extraction even at ultra-low inputs.

To analyse the efficiency of our method to exclude RNA fragments larger than RPFs (more than 36 nt), we digested total RNA from a human myelogenous leukaemia cell line (K562) with micrococcal nuclease (MNase), purified the sample and subjected it to Ribo-ITP (Fig. 2b). We achieved 94% exclusion of the unwanted large RNA fragments (more than 36 nt) (Fig. 2b). Finally, to verify the ability of Ribo-ITP to extract RNAs from complex cellular lysates, we spiked RPF-sized synthetic RNAs (17, 21, 25 and 29 nt) into total cellular lysates from approximately 1,000 K562 cells. Ribo-ITP of this sample recovered the spiked RNAs with stringent size selection and high yield (Extended Data Fig. 1g,h). Collectively, these results indicate that Ribo-ITP can simultaneously extract and size select RPF-size RNAs from cellular lysates with high yield.

## Ribo-ITP single-cell ribosome occupancy

**Extended Data Fig. 2:**
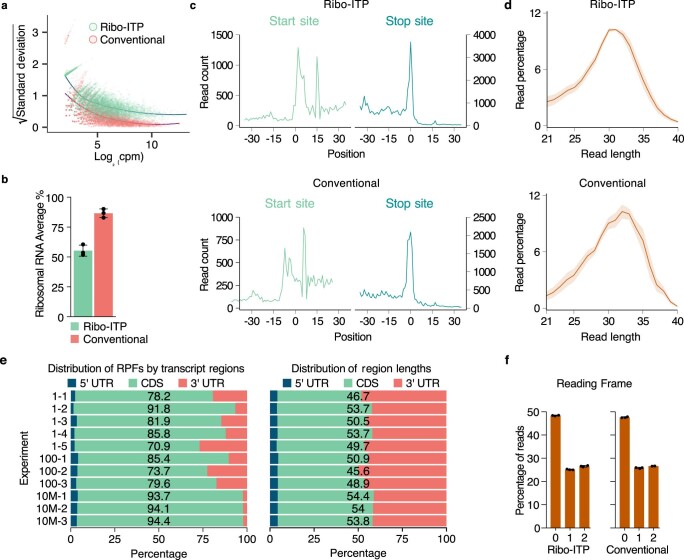
Comparison between Ribo-ITP and conventional ribosome profiling. Ribo-ITP experiments from ~100-cells were compared to the conventional ribosome profiling from ~10M cells for all panels. **a**, Transcripts with at least one count per million (cpm) in at least two out of three replicates were selected. Log_2_ of mean cpm (x-axis) was plotted against the square root of the standard deviation of cpm (y-axis). Green (Ribo-ITP) and red (conventional) points represent individual transcripts with mean log_2_(cpm) > 2. **b**, The percentage of clipped reads that mapped to ribosomal RNAs was calculated using RiboFlow^61^. The mean and its standard error were shown (n = 3; see also Supplementary Table 1). **c**, In the metagene plots, position 0 corresponds to the start (left, light green) or stop (right, dark green) site. Ribosome footprints were adjusted according to their A-site offsets. One representative replicate for each method is plotted. **d**, The mean percentage of specific read lengths among the total mapped reads is plotted. Ribbons around the lines represent standard error of the mean. **e**, The percentage of ribosome profiling reads mapped to the CDS is indicated for each experiment (left). Single cell data from K562 cells were generated using either RNase I digestion (1-1, 1-2, 1-3) or MNase (1-4, 1-5). The sum of weighted counts across transcripts were plotted for comparison (right). The weighted sum was calculated for each transcript by multiplying its region length by the total number of ribosome footprints. **f**, Ribosome footprints from Ribo-ITP and the conventional method were used to calculate the mean fraction of reads mapping to each reading frame (Methods). Error bars indicate the standard error of the mean of replicates.

To validate the quality of ribosome profiling data, we performed Ribo-ITP from single and 100 K562 cells as well as conventional ribosome profiling using the gold-standard method of monosome isolation^17^ from 10 million K562 cells (Fig. 2c). Ribosome occupancy measurements from 100 cells obtained using Ribo-ITP were highly reproducible across replicates (Fig. 2d, Extended Data Fig. 2a,b and Supplementary Table 1). The footprints displayed enrichments at annotated translation start and stop sites (Extended Data Fig. 2c,d). The majority of transcript mapping reads originated from the coding sequences (CDS) and displayed 3-nt periodicity that was highly enriched over the distribution expected from random fragmentation (Chi-squared test, *P* < 2.2 × 10^−16^; Extended Data Fig. 2e,f). Critically, ribosome profiling measurements from 100 cells generated by Ribo-ITP recapitulated the conventional ribosome profiling measurement (Spearman correlation coefficient of 0.88; *P* < 2.2 × 10^−16^; Fig. 2d). These results reveal that ribosome occupancy can be accurately measured from as few as 100 human cells using Ribo-ITP.

**Fig. 3:**
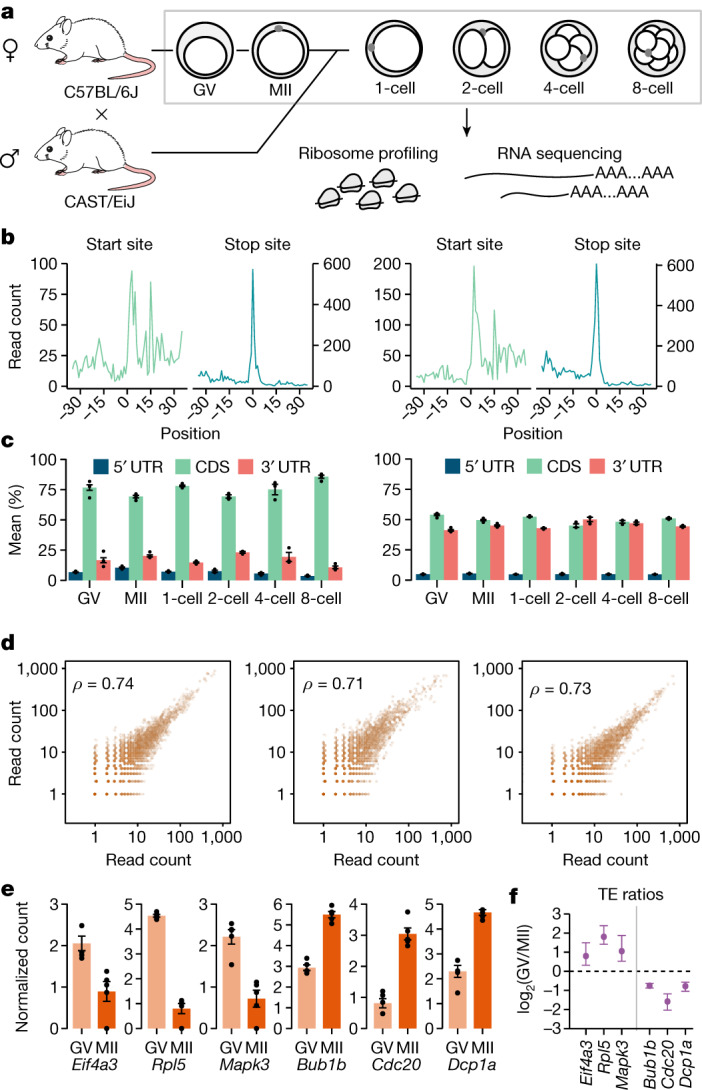
Ribo-ITP enables single-cell and single-embryo measurements of ribosome occupancy. **a**, Schematic of the mouse experiments. Unfertilized oocytes (germinal vesicle (GV) and MII stage) from the C57BL/6J strain along with zygotes to the eight-cell-stage embryos from a crossbreed of two strains (C57BL/6J and CAST/EiJ) were collected for RNA expression and ribosome occupancy measurements. **b**, Ribosome occupancy around the translation start and stop sites in a representative zygote (one cell; left) and an eight-cell-stage embryo (right). Translation start (or stop) sites are denoted by the position 0. Aggregated read counts (*y* axis) relative to the start (or stop) sites are plotted after A-site correction (Methods). **c**, Distribution of reads across transcript regions (5′ UTR, CDS and 3′ UTR) are shown (left). The distribution of the lengths of these regions weighted by their ribosome occupancy are also depicted (right). The error bars indicate the standard error of the mean percentages. **d**, Pairwise correlation of gene-level ribosome occupancy in single cells (GV mouse oocyte (left), MII mouse oocyte (middle) and one-cell mouse embryo (right)) are plotted along with Spearman correlation coefficients (top left). **e**, The standard error and mean of the centred log ratio of the ribosome occupancy (*y* axis) were plotted for representative transcripts that were previously shown to have increased polysome association in GV-stage (**e**) or MII-stage (**f**) oocytes^18^ (remaining genes are shown in Supplementary Fig. 12). **f**, Translation efficiency was calculated by dividing ribosome occupancy by RNA expression for germinal vesicle-stage and MII-stage oocytes. For the selected transcripts, the log ratio of translation efficiency between these two stages is plotted along with the standard error of the mean across replicates.

Next, we applied Ribo-ITP and RNA sequencing (RNA-seq) to characterize the translation changes of single oocytes at germinal vesicle and metaphase II (MII) stages and single embryos from the one-cell zygote to eight-cell stages in mice (Fig. 3a and Supplementary Table 1). In particular, the initial division of zygotes occurs in the absence of new RNA synthesis, rendering the translation of stored maternal transcripts absolutely essential for the early stages of development.

**Extended Data Fig. 3:**
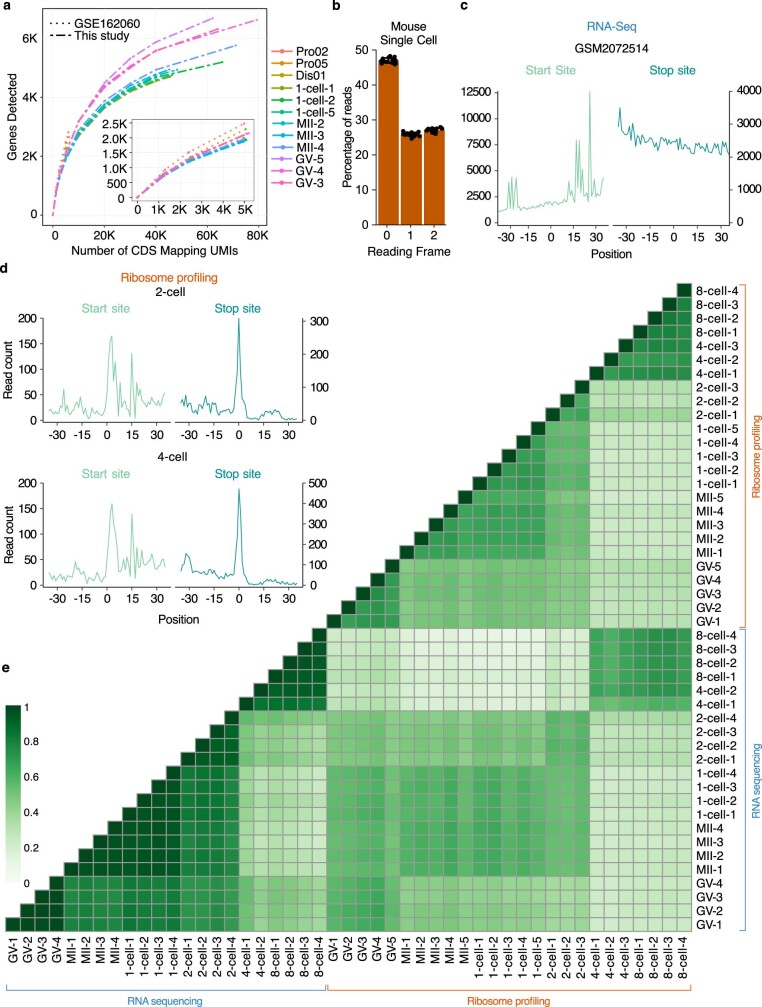
Quality Control for single cell and single embryo measurements of ribosome occupancy and RNA expression. **a**, The number of reads (x-axis) is plotted against the number of detected genes (y-axis). Three representative replicates are shown for each stage of development used for single cell ribosome profiling experiments and from GSE162060 (Pro02_TCGTCAGTAC, Pro05_CCAATCCAGG and Dis01_TCGCTCTGCT). The three mouse cells highlighted from GSE162060 were selected to represent median coverage in that study. The total number of detected genes using all CDS mapping reads is indicated along with genes detected with 1.25k, 2.5k, 5k, 10k, 20k, 30k and 40k sub-sampled coding regions mapping UMIs. The inset plot shows the zoomed-in version of the same data in the x-axis from 0 to 5k. **b**, Ribosome footprints from n = 15 single cell Ribo-ITP samples from GV, MII and 1-cell stages were used to calculate the fraction of reads mapping to each reading frame (Methods). Error bars indicate the standard error of the mean of replicates. **c**, Metagene plots of translation start and stop sites from a representative RNA-Seq experiment. In contrast to the ribosome profiling data, there is no detectable peak observed at translation start or stop sites. **d**, Metagene plots of translation start and stop sites from a representative Ribo-ITP experiment using a 2-cell or a 4-cell stage mouse embryo. Start sites (light green, left) and stop sites (dark green, right) are at position 0 on the x-axis. Positions of the aligned reads are adjusted according to their A-site offsets. **e**, CDS-mapping read counts from each transcript were used to compute the Spearman correlation coefficient. Ribo-ITP (orange) and RNA-Seq (blue) experiments are ordered by developmental stage. The colors indicate the strength of the correlation.

In our single-cell ribosome occupancy data—the germinal vesicle, MII and one-cell stages—we observed a median of 48,017 unique molecules originating from the coding regions of transcripts, leading to the detection of an average of 5,064 genes per cell (range 4,076–6,679; Extended Data Fig. 3a,b). Single-oocyte and single-embryo ribosome profiling data demonstrated the expected enrichment of footprints mapping to coding regions and characteristic enrichments at the start and stop sites (Fig. 3b,c and Extended Data Fig. 3c,d). Replicate measurements of ribosome occupancy were highly correlated (Fig. 3d and Extended Data Fig. 3e).

**Extended Data Fig. 4:**
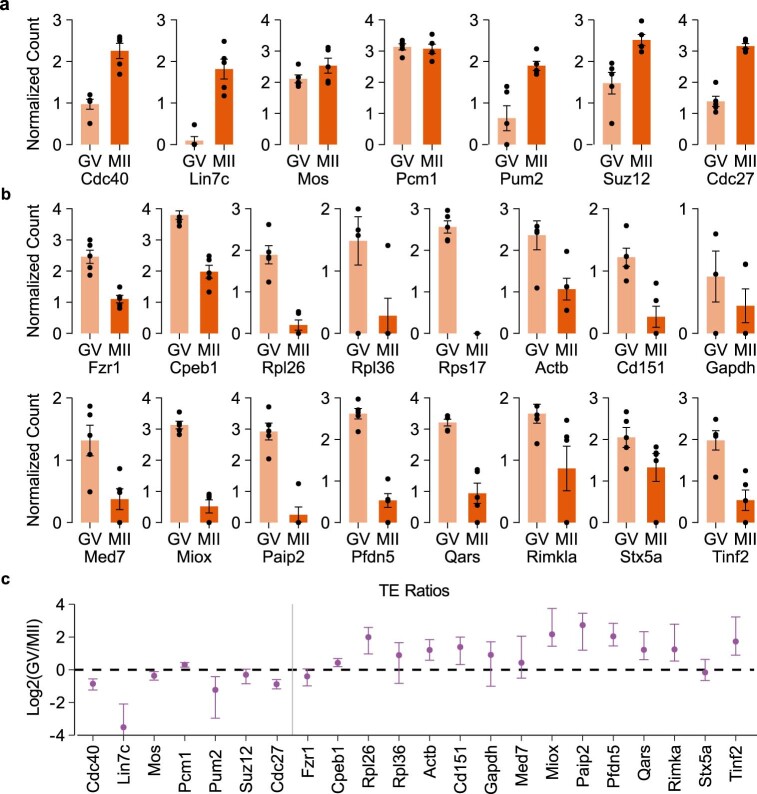
Ribosome occupancy in GV- and MII-stages of transcripts with previously identified differential polysome association. **a**, The mean of centered log-ratio of ribosome occupancy (y-axis) was plotted along with the standard error of the mean. These transcripts were identified as having increased polysome association in the MII-stage compared to GV-stage^18^. **b**, Similar to panel a with the exception that these transcripts were found to display decreased polysome association in the MII-stage compared to the GV-stage^18^. **c**, log_2_ ratio of translation efficiencies (ribosome occupancy/RNA expression) for the highlighted genes in GV compared to MII stage oocytes are plotted along with their standard error. The vertical bar separates genes in panel **a** from **b**.

To validate the quality of our single-cell ribosome profiling measurements, we compared our results to a previous study that collected approximately 500–600 germinal vesicle-stage and MII-stage oocytes and validated changes in polysome association with quantitative  reverse transcription–PCR experiments for 29 transcripts^18^. Our single-cell ribosome profiling measurements recapitulated the previously identified changes in ribosome association for 28 out of 29 RNAs (Fig. 3e and Extended Data Fig. 4). Together, our results indicate that Ribo-ITP enables highly consistent and high-quality ribosome occupancy measurements from single cells and single embryos during early mouse development.

## Ribo-ITP allele-specific translation

In mouse development, we currently do not know when zygotically synthesized RNAs engage with ribosomes and whether there exist any gene-specific and allele-specific differences in these dynamics. We first addressed the question of allele-specific expression following zygotic genome activation. Both deterministic and stochastic differences in allele expression ratios are believed to contribute to differentiation and normal development, although studies have been limited to the level of epigenetics and transcription in the early mouse embryo^19,20^.

To distinguish RNA molecules derived from the maternal and paternal alleles, we analysed embryos from a cross of two mouse strains (C57BL/6J × CAST/EiJ). Using strain-specific single-nucleotide polymorphisms (SNPs) to distinguish maternal and paternal RNAs, we detected 229,991 unique parent-of-origin-specific RPFs mapping to coding regions (Methods).

**Fig. 4:**
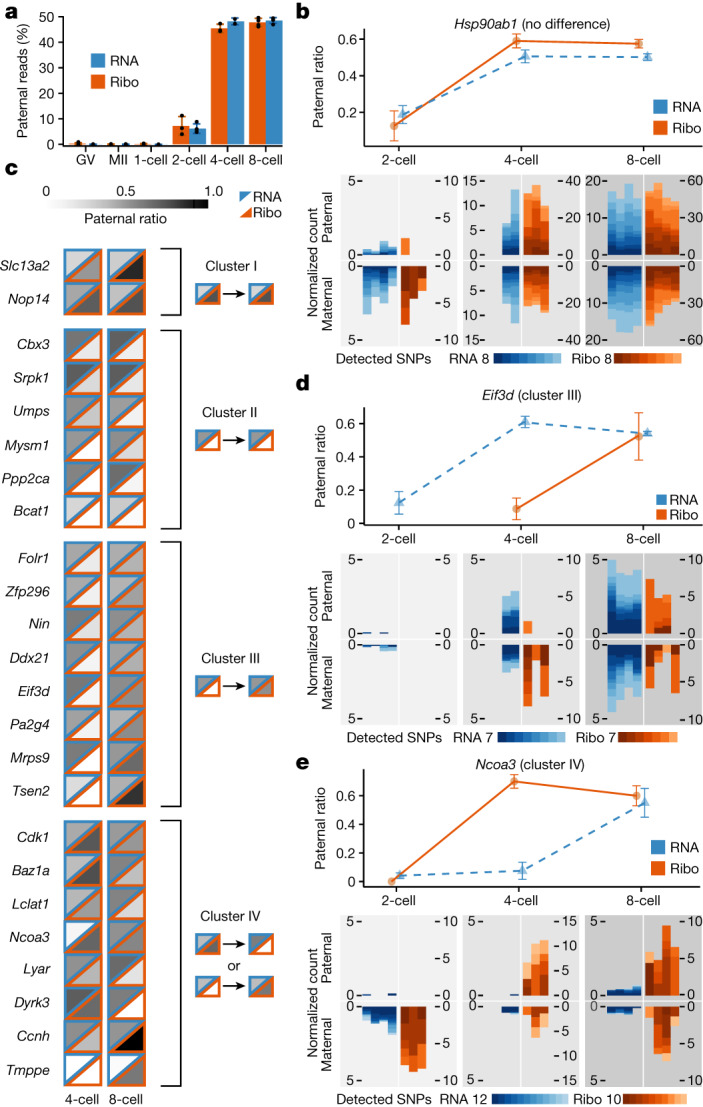
Allele-specific translation and RNA expression in early mouse development. **a**, Strain-specific SNPs were used to assign sequencing reads to the paternal and maternal allele (Methods). The standard error and mean of the percentage of paternal reads in each stage (*y* axis) are plotted. **b**,**d**,**e**, Line plots (top) indicate the percentage of paternal reads (*y* axis) in RNA-seq and Ribo-ITP experiments. The reads are combined across replicates, and error bars indicate the standard error of the mean of paternal ratios. Maternal and paternal read counts per 10,000 are also plotted for all individual replicates and SNPs (bottom). The total number of detected coding SNPs and their corresponding colours are shown with colour scales. **c**, The percentage of paternal reads is indicated by different shades of grey for ribosome occupancy (lower triangles with orange borders) and RNA expression (upper triangles with blue borders). Genes that displayed differential ribosome engagement in an allele-specific manner compared with RNA expression were grouped into four clusters. The prototypical shared pattern in each cluster is displayed on the right.

**Extended Data Fig. 5:**
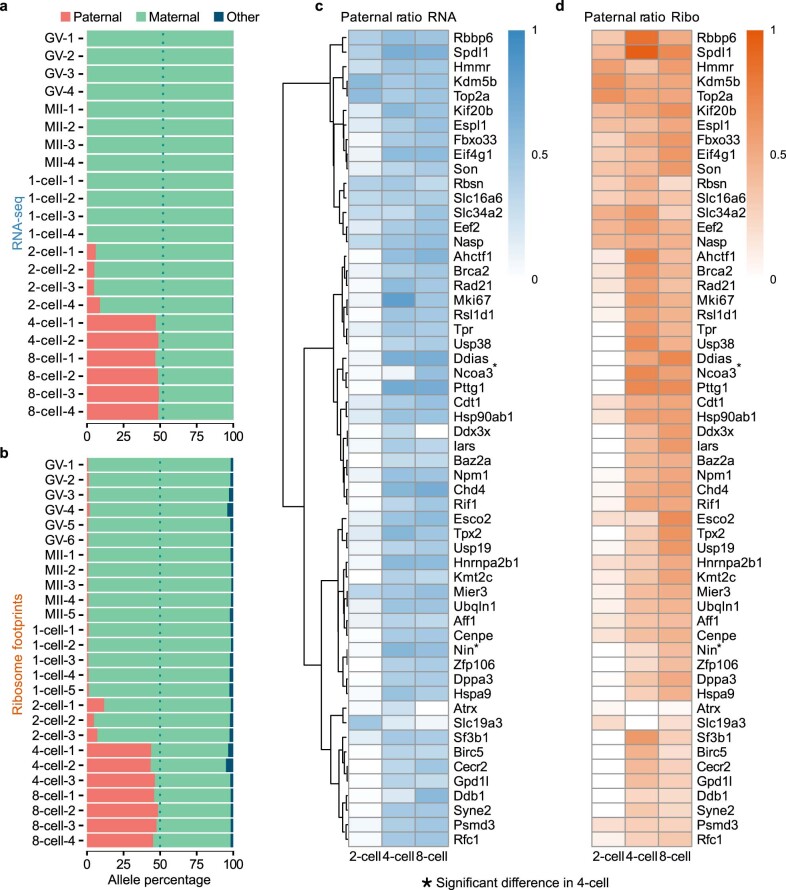
Allele-specific ribosome occupancy and RNA expression. **a**, Reads from RNA-seq experiments that overlap strain-specific SNPs were used to determine the percentage of reads that match the maternal (green) and paternal (red) allele. A small percentage of reads differed from either allele and are labeled as “other” (dark blue). **b**, Ribosome footprints from Ribo-ITP experiments were used as in panel a. **c**, The percentage of RNA sequencing reads that originate from the paternal allele was visualized for genes with at least 10 allele-specific reads at each stage. **d**, The percentage of ribosome footprints that were assigned to the paternal allele was plotted for the set of genes in panel c.

To monitor allele-specific ribosome engagement alongside corresponding RNA expression^21^, we specifically focused on the paternal allele, which, unlike RNA of maternal origin, is a proxy of newly synthesized transcripts (Methods). We analysed the global pattern of ribosome engagement of paternally derived RNAs, that is, paternal allele ratios, by aggregating reads across all detected genes. We found that, coinciding with the activation of zygotic transcription, the paternal ratio of ribosome occupancy steadily increased from 7.1% in the two-cell-stage embryos to 47.7% in the eight-cell-stage embryos (Fig. 4a and Extended Data Fig. 5a,b). We discovered that the ratio of paternal alleles across these stages was statistically indistinguishable between ribosome occupancy and RNA expression (*t*-test; *P* > 0.14 for all stages; Fig. 4a). This result indicates that ribosome engagement is overall concurrent with the synthesis of paternal RNAs via zygotic genome activation.

**Extended Data Fig. 6:**
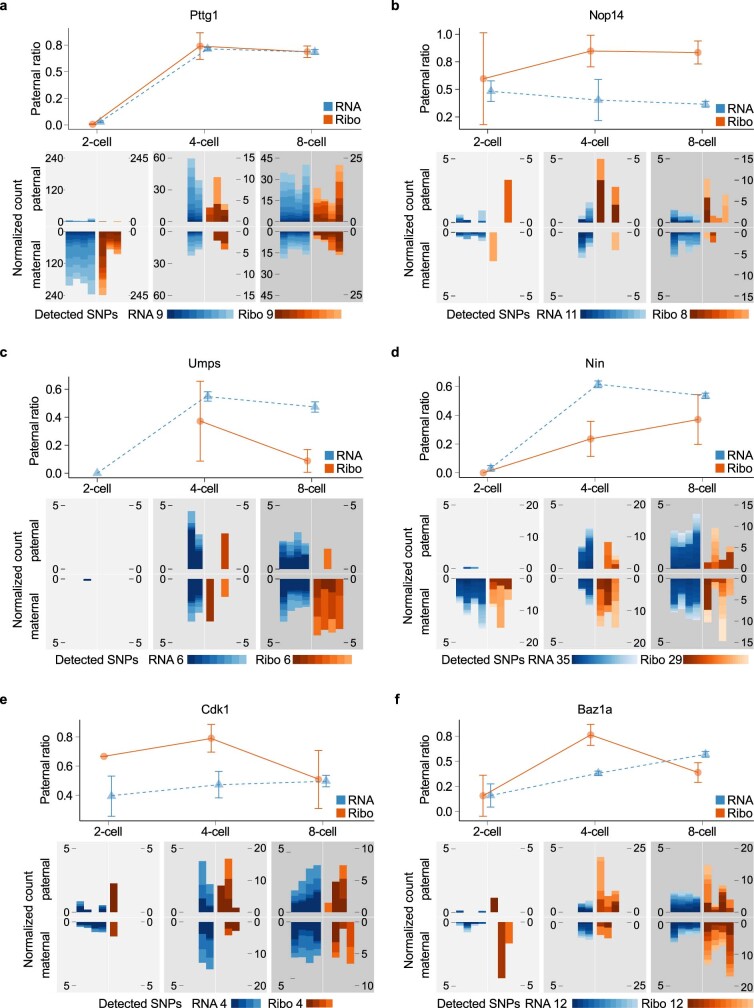
Representative genes with allele-specific difference in ribosome occupancy and RNA expression. Line-plots (top) indicate the mean percentage of paternal reads (y-axis) in RNA-Seq and Ribo-ITP experiments. The reads are combined across replicates and error bars indicate standard error of the mean of paternal ratios. At the bottom, maternal and paternal reads counts per 10k are plotted for all individual replicates and SNPs. The total number of detected coding SNPs and their corresponding colors are shown with color scales. Each vertical bar corresponds to a replicate experiment. **a**, Representative gene with no difference in allele- specific ribosome occupancy compared to RNA expression. **b**, Representative gene from Cluster I (Fig. 4). **c**, Representative gene from cluster II (Fig. 4). **d**, Representative gene from cluster III (Fig. 4) with delayed engagement of ribosomes in allele- and stage-specific manner i.e. parental RNA expression is observed at or before the 4- cell stage, yet these RNAs predominantly engage with ribosomes only in the 8-cell stage. **e**,**f**, Representative genes from Cluster IV (Fig. 4).

We next considered whether there are any gene-specific exceptions to the observed global pattern of equal allelic ratios in RNA expression and ribosome occupancy in the early mouse embryo. As expected, the majority of genes exhibited a similar ratio of paternal reads in both RNA expression and ribosome occupancy (Extended Data Fig. 5c,d). An example gene, *Hsp90ab1*, had eight distinct coding SNPs differentiating the two alleles across multiple replicates in RNA-seq and Ribo-ITP. The high similarity of paternal allele ratios in RNA expression and ribosome occupancy was consistently observed for multiple replicates and supported by distinct SNPs (Fig. 4b and Extended Data Fig. 6a).

We also identified 24 genes that had differential ribosome engagement in an allele-specific manner compared with RNA expression (two-sample test for the equality of proportions; Methods). These 24 genes were clustered into four groups based on the patterns of allele-specific expression (Fig. 4c). Although cluster I and cluster II encompass genes that display consistent allele-specific ribosome occupancy bias throughout early development (Extended Data Fig. 6b,c), genes in the other two clusters displayed allele-specific ribosome occupancy in a stage-dependent manner.

In particular, several genes including *Eif3d* displayed delayed engagement of newly transcribed paternal RNA with ribosomes. Specifically, the paternal allele was robustly expressed in four-cell embryos, yet ribosome association of the paternal allele was delayed until the eight-cell stage (Fig. 4d and Extended Data Fig. 6d (cluster III)). This observation suggests that specific transcripts may either have slow kinetics of nuclear export or are sequestered in translationally inactive compartments until their subsequent association with ribosomes occurs in the eight-cell stage.

Genes in the last group (cluster IV) included *Cdk1*, a key regulator of the cell cycle, and *Baz1a*, a chromatin remodelling factor (Fig. 4e and Extended Data Fig. 6e,f). Together, our results reveal that for most transcripts, ribosome engagement is concurrent with zygotic activation and paternal RNA expression. Yet, a small number exhibit allele-specific ribosome engagement during different stages.

**Extended Data Fig. 7:**
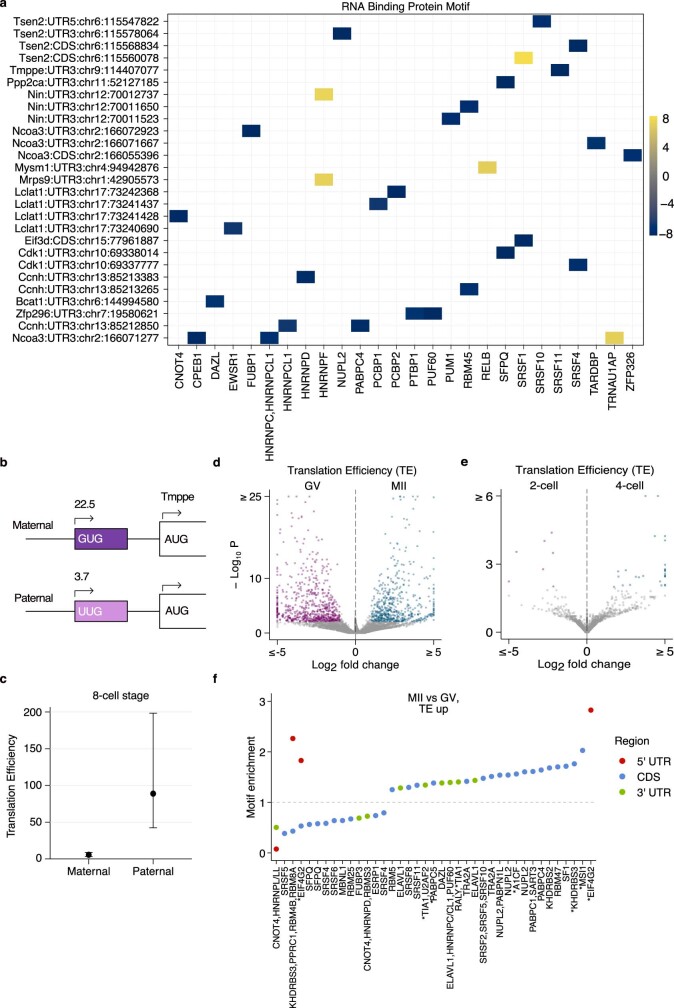
Differential translational efficiency and enrichment of RBP motifs across stages in oocyte and embryonic development. **a**, The oRNAment matrix similarity score^56^ was computed for maternal and paternal SNPs that intersect RBP motifs, and the max absolute difference in scores was determined. SNP-motifs in the 95th percentile of the absolute differences are shown. A positive difference (yellow) indicates the paternal SNP more closely matches the RBP motif, while a negative difference (blue) indicates a better match for the maternal SNP. RBPs with a shared motif are comma-delimited. SNP coordinates are relative to the mm10 build. **b**, Schematic of a predicted upstream open reading frame (uORF) in *Tmppe*. uORF value and intensity of fill indicate the efficiency of the corresponding non-canonical translation initiation sites from mouse PD-31 cells^79^. **c**, The mean ratio of allele-specific reads in ribosome profiling (n = 4) to RNA-seq (n = 4) was plotted after aggregating data across SNPs and experiments. Each experiment is normalized to 10k SNP-informative reads to account for differences in sequencing depth. The error bars correspond to the 80% confidence interval calculated using bootstrap sampling of replicates. A pseudocount of 0.5 was added to both normalized RNA-seq and ribosome profiling counts. **d,e,** Volcano plots depict the statistical significance (y-axis) and log_2_ fold-change (x-axis) in translation efficiency between the GV- to MII-stage oocytes and between the 2-cell to 4-cell stage embryos. The purple and blue points indicate transcripts with a significant difference in translation efficiency between the compared stages (FDR < 0.01). **f**, Enrichment and depletion of RBP motifs in genes with increased TE between MII and GV stages was determined by Transite^55^ (Benjamini-Hochberg adjusted p-value < 0.0001, Supplementary Table 5) and annotated with oRNAment^56^ RBPs. RBPs that share the same consensus motif are comma-delimited, and RBPs with no detectable expression are marked with an asterisk.

To uncover potential genetic mechanisms of allele-specific translation efficiency, we determined SNPs that are predicted to alter RNA-binding protein (RBP) motifs or other potential translation regulatory sequences (Extended Data Fig. 7a–c and Supplementary Table 2). We identified 27 SNPs (13 out of 24 genes in Fig. 4c) that altered RBP motifs in an allele-specific manner. These included changes in binding motifs of several translational regulators (DAZL, CPEB1 and PUM1) previously implicated in early mouse embryonic development^18,22–26^. We also identified allele-specific motifs for SRSF1 that are associated with higher ribosome occupancy in *Tsen2* and *Eif3d*. Our RNA expression and ribosome profiling data revealed that SRSF1 is robustly expressed in early embryos. Given the established role of SRSF1 as a translational activator in other contexts^27^, our data suggest this RBP as a potential translation regulator in early mouse development. Furthermore, a SNP in the 5′ untranslated region (UTR) of *Tmppe* was predicted to support translation of an upstream open reading frame (uORF) on the C57BL allele (Extended Data Fig. 7b). We observed lower ribosome occupancy of *Tmppe* from the C57BL allele consistent with the known inhibitory role of uORFs^28^. Together, these results suggest that a multitude of mechanisms probably underlie differential allele-specific ribosome engagement, including changes to RBP binding.

## Differential translation efficiency during development

**Fig. 5:**
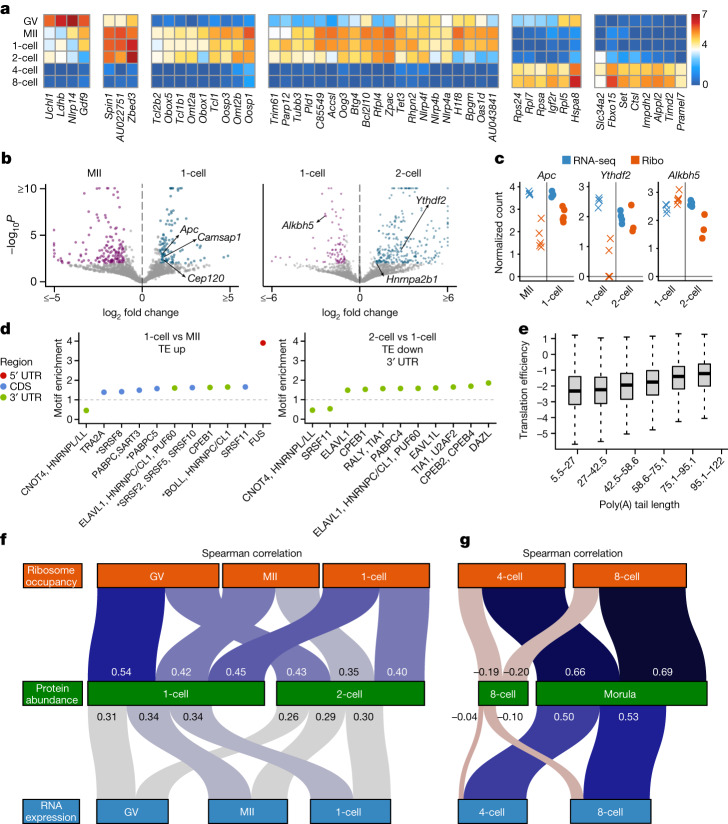
Differential translation efficiency between developmental stages and association between ribosome occupancy and protein abundance. **a**, Fifty genes with the highest variability in ribosome occupancy across developmental stages are plotted (Methods). The colours correspond to the mean of the centred log ratio of ribosome occupancy. **b**, Volcano plots depict the statistical significance (*y* axis) and log_2_ fold change (*x* axis) in translation efficiency between two developmental stages (Methods). Coloured points indicate transcripts with significant differences (false discovery rate of less than 0.01). **c**, The centred log-ratio normalized read counts from Ribo-ITP and RNA-seq experiments are plotted for the highlighted genes. All replicate measurements from the given developmental stage are shown. **d**, Enrichment and depletion of RBP motifs were determined by Transite^55^ (Benjamini–Hochberg *P* < 0.0001; Supplementary Table 5) and annotated with oRNAment^56^ RBPs. RBPs that share the same consensus motif are comma-delimited, and RBPs with no detectable expression are marked with an asterisk. TE, translation efficiency. **e**, Transcripts were grouped by their mean poly(A) tail length in the zygote into six equal-sized bins. The distribution of their corresponding translation efficiencies (Methods) is visualized using boxplots. The horizontal line corresponds to the median, the box represents the interquartile range and the whiskers extend to 1.5 times the interquartile range. **f**,**g**, Sankey diagrams depict the relationships between protein abundance with RNA expression and ribosome occupancy. The colour and thickness of the links connecting the nodes are proportional to the strength of the corrected Spearman rank correlation (Methods).

We next characterized transcript-specific changes in translation across the studied developmental stages. Transcripts with the highest variability of ribosome occupancy revealed two major transitions: one between germinal vesicle-stage and MII-stage oocytes and another between two-cell-stage and four-cell-stage embryos (Fig. 5a). We then focused on identifying the set of transcripts with differences at the translational level, that is, with differential translation efficiency, as defined by significant changes in ribosome occupancy while controlling for RNA abundance (Methods).

We uncovered a large number of genes that exhibited translation efficiency changes during oocyte maturation from germinal vesicle to MII stage, as well as upon fertilization (Fig. 5b, Extended Data Fig. 7 and Supplementary Table 3). Among the 129 genes that were translationally upregulated upon fertilization, 126 had no statistically significant changes in RNA expression (false discovery rate of less than 0.01; one-cell embryo versus MII-stage oocyte; Fig. 5b,c). These genes were significantly enriched for cytoskeleton organization (Fisher’s exact test odds ratio of 4.67; *P* = 8.5 × 10^−9^; Supplementary Table 4), and include *Apc* along with several other genes involved in centrosome organization (for example, *Cenpe*, *Cep120*, *Camsap1* and *Numa1*). The first mitotic division in mammals is both longer and more error-prone than somatic mitotic events^29^. Furthermore, fertilization demarcates the beginning of a transition from multipolar acentrosomal division to the typical bipolar spindles organized by the centrosomes^30^. Critically, *Apc* activation is required for this reorganization and the dynamics of its activation underlies the prolonged first mitosis of mouse embryos^31^. Our results reveal differential translation efficiency as a key regulatory mechanism of this class of transcripts in the absence of any transcription.

**Extended Data Fig. 8:**
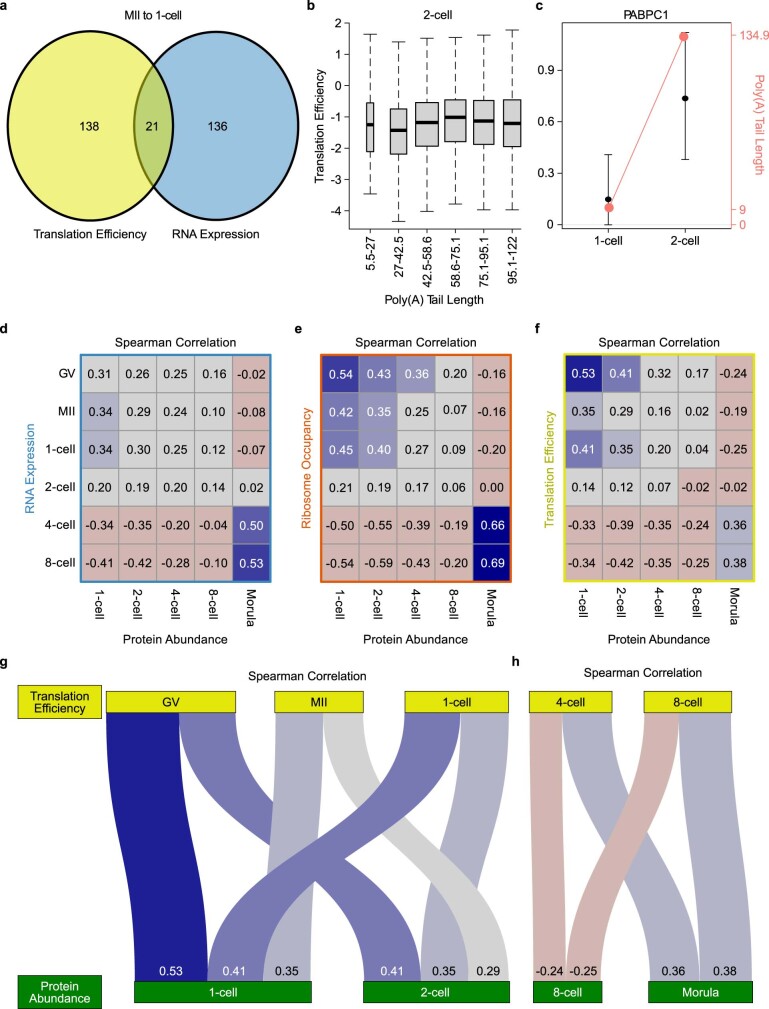
Relationship between RNA expression, translation and protein abundance across embryonic stages. **a**, Venn diagram of the overlap between the sets of transcripts that are reduced in RNA abundance or translational efficiency upon fertilization (MII-oocyte to the zygote transition). **b**, The mean poly(A) tail length and translation efficiency was calculated using data from 2-cell stage embryos (n = 3 Ribo-ITP; n = 4 RNA-Seq; see Methods). The boxes correspond to interquartile range and the horizontal lines indicate the median. **c**, Mean translation efficiency and its 90% confidence interval are plotted for PABPC1 along with its mean poly(A) length in 1-cell (n = 5 Ribo-ITP; n = 4 RNA-Seq) and 2-cell embryos. **d,e,f**, Pairwise correlation between protein abundance and **d**, RNA expression or **e**, Ribosome occupancy or **f**, Translation efficiency. Spearman correlation coefficient with Spearman’s correction is reported inside the heatmaps. **g,h**, Sankey diagrams depict the relationships between translation efficiency and protein abundance. The color and the thickness of the paths connecting the nodes are proportional to the correlation coefficient.

Next, we considered the relationship between translation regulation and RNA stability in this developmental transition. Given that no new transcription takes place, any reduction in RNA expression can specifically be attributed to degradation. We found a significant overlap between RNAs that are significantly reduced in expression and RNAs translationally downregulated in the zygote (Fisher’s exact test odds ratio of 5.87; *P* = 2.24 × 10^−9^; Extended Data Fig. 8), suggesting that these two gene expression modalities may function synergistically.

When we compared two-cell embryos to the zygote, we found a significant enrichment for RBPs among genes that had increased translational efficiency (Fisher’s exact test odds ratio of 2.93; *P* = 3.1 × 10^−10^; Supplementary Table 3). These include three genes that function as ‘readers’ of *N*^6^-methyladenosine RNA modifications (*Hnrnpa2b1*, *Ythdf2* and *Ythdc1*; Fig. 5b,c). Recent work has revealed all three of these genes to be required for successful early embryonic development^32–35^. Maternal depletion of *Ythdf2* in mice causes cytokinesis defects and arrest at the two-cell stage^33^. Similarly, reduced *Hnrnpa2b1* expression delayed embryonic development after the four-cell stage^32^. A recent analysis of *Hnrnpa2b1* expression during preimplantation development had revealed negligible differences in RNA expression between the zygote and two-cell mouse embryos, despite a dramatic increase in its protein abundance in two-cell embryos^32^. Our analyses suggest that enhanced translation of *Hnrnpa2b1* is probably responsible for this observation. Although *N*^6^-methyladenosine ‘readers’ displayed increased translation efficiency in two-cell embryos, the key demethylase that removes *N*^6^-methyladenosine, *Alkbh5* (ref. ^36^), was one of the most significantly downregulated genes in terms of translation efficiency (Fig. 5c; adjusted *P* = 8.4 × 10^−8^). Together, our results reveal translational regulation as a shared mode of co-regulation of genes involved in *N*^6^-methyladenosine modification of RNAs.

To explore potential mechanisms associated with differential translation efficiency, we carried out an unbiased analysis for enrichment or depletion of heptamer motifs in differential genes (Methods; Supplementary Table 5). Several of the heptamers matched a RBP consensus motif and displayed consistent enrichment or depletion in differential genes across stages, suggesting their involvement in shaping expression during early development (Fig. 5d, Extended Data Fig. 7f and Supplementary Table 5). In particular, we discovered that transcripts with DAZL-binding sites within their 3′ UTRs were translationally downregulated in the transition from zygote to the two-cell stage (Fig. 5d). DAZL has previously been implicated as a translational activator during gametogenesis^18,22,37^. The one-cell to two-cell embryonic transition demarcates a marked reduction in DAZL abundance coinciding with the translational downregulation of genes with DAZL-binding sites in their 3′ UTRs^18,22^. These findings suggest that DAZL may also have an important regulatory role in the context of preimplantation development in addition to its known role during gametogenesis.

Changes in poly(A) tail length is an important regulatory mechanism controlling translation efficiency during meiotic maturation of mouse oocytes^38^ and early development of *Xenopus* and zebrafish^39,40^. We used TAIL-seq measurements in mouse embryos from one-cell and two-cell stages^41^, and found that poly(A) tail length is significantly associated with translation efficiency in the zygote (Fig. 5e; Spearman rank correlation of 0.32; *P* < 2.2 × 10^−16^). However, the coupling between poly(A) tail length and translation efficiency is completely lost by the two-cell stage coinciding with the activation of the zygotic genome (Extended Data Fig. 8b; Spearman rank correlation of 0.001; *P* = 0.9; Extended Data Fig. 10). Previous work^40^ had postulated that poly(A) tail length would be expected to regulate translation in systems in which transcription is repressed and cytoplasmic polyadenylation is active. Our analysis validates this prediction in a mammalian organism, consistent with findings in zebrafish and frog embryos^40^.

To explore why the strong coupling between poly(A) tail length and translation efficiency breaks down in later stages of development, we analysed PABPC1 expression, which mediates the effect of poly(A) tail length on translation^42^. We found that PABPC1 is itself regulated translationally and that its mean poly(A) tail is markedly extended in two-cell-stage embryos compared with zygotes (Extended Data Fig. 8c). These findings implicate the limiting presence of PABPC1 in zygotes as a potential explanation for the coupling between poly(A) tail length and translation efficiency, consistent with findings in *Xenopus* oocytes^43^. Collectively, our results indicate a conserved role for poly(A) tail length in dictating translation efficiency in the early stages of mouse embryogenesis.

## Translation efficiency contribution to protein abundance

The proteome of the zygote is composed of maternally deposited proteins and those newly synthesized after fertilization^1^. Here we assessed the contribution of translation in determining protein abundance using mass spectrometry measurements from approximately 8,000 embryos from each stage of mouse preimplantation development^6^. Out of more than 5,000 genes detected in our single-embryo ribosome profiling and RNA-seq experiments, 3,287 genes had been quantified at the protein level^6^.

**Extended Data Fig. 9:**
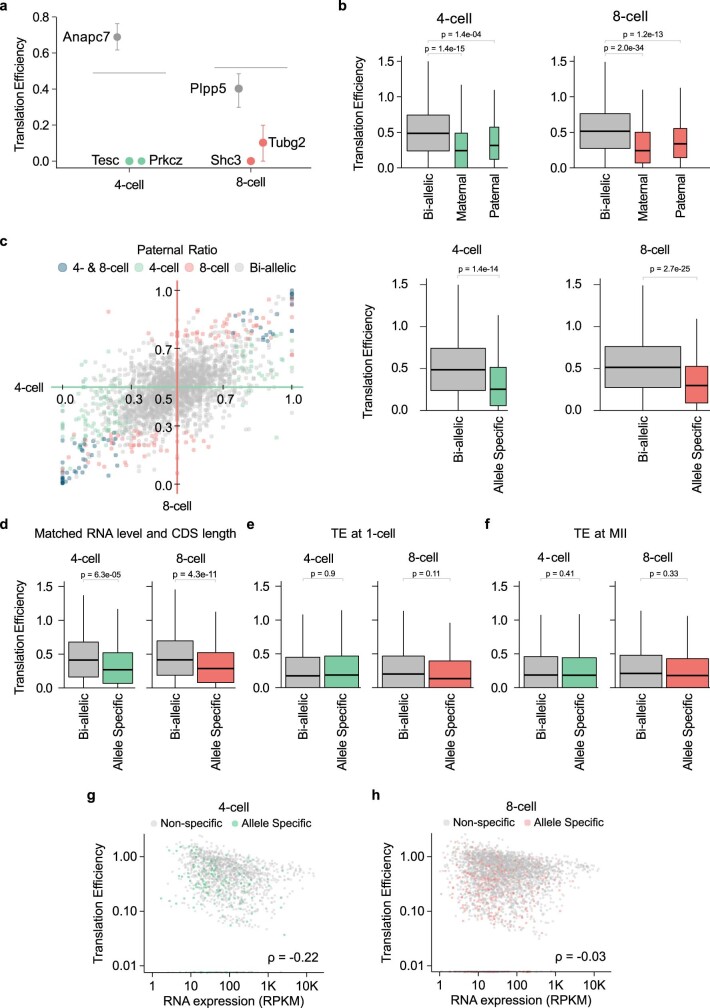
Translation efficiency of genes with allelic-biased RNA expression. For all plots, boxes correspond to interquartile range and the horizontal lines indicate the median. The whiskers extend from the hinge to at most 1.5 times the interquartile range. P-values are calculated using the two-sided Wilcoxon rank sum test. Translation efficiency is defined by average ribosome occupancy (MII n = 5, 1-cell n = 5, 4-cell n = 3, 8-cell n = 4) divided by average RNA expression (MII n = 4, 1-cell n = 4, 4-cell n = 2, 8-cell n = 4) **a**, Translation efficiency is plotted for two bi-allelic genes (*Anapc7* and *Plpp5*), two maternally biased genes (*Tesc* and *Tubg2*) and two paternally biased genes (*Prkcz* and *Shc3*). Vertical bars indicate 95% confidence intervals of bootstrap sample based variability in translation efficiency. Horizontal lines depict the median translation efficiency of all bi-allelic genes. **b**, Translation efficiency of unbiased (bi-allelic), paternally and maternally biased genes are plotted. **c**, We define a high-confidence set of allelic-biased transcripts that are supported by more than one SNP (Methods). The scatter plot, on the left, shows the paternal ratios of the genes in 4-cell (x-axis) and 8-cell (y-axis) stages. Genes are colored by their allelic bias and unbiased genes are colored in gray. **d**, A nearest neighbor matching method was used to select a subset of bi-allelic genes with matched distribution of RNA expression and CDS length compared to the allele-specific genes (Methods). **e,f**, The genes with allele-biased expression at the 4-cell and 8-cell stages were compared to other genes with respect to their translation efficiency at the 1-cell and MII stages. The bi-allelic genes were matched to allele-specific ones with respect to RNA expression levels and CDS lengths. **g,h** For each transcript, the x-axis represents the mean RPKM in RNA-seq and the y-axis is the corresponding mean translation efficiency. Genes with allelic expression bias are colored.

We found that the zygotic proteome is only modestly correlated with RNA expression of the zygote (Spearman rank correlation of 0.34; *P* < 2.2 × 10^−16^), in agreement with previous work that reported weak correlation between RNA expression and protein abundance^6,44^. By contrast, zygotic protein abundance is significantly better correlated with zygotic ribosome occupancy and translation efficiency than RNA expression (Spearman rank correlations of 0.45 and 0.41 versus 0.34; *P* < 2.2 × 10^−16^; Fig. 5f and Extended Data Fig. 9d–f).

Critically, we discovered that translation efficiency of the germinal vesicle-stage oocytes had the strongest relationship with the zygotic protein abundance (Fig. 5f and Extended Data Fig. 9d–g; Spearman rank correlation of 0.53, *P* < 2.2 × 10^−16^). This key contribution is undetectable at the level of RNA expression as RNA abundance in germinal vesicle-stage oocytes is much more weakly associated with zygotic protein abundance (Spearman rank correlation of 0.31; *P* < 2.2 × 10^−16^). Our results reveal that maternal translation is the predominant contributor to the zygotic proteome.

The coupling of rapid degradation of maternally deposited RNAs and the onset of zygotic transcription fundamentally remodels the RNA content of the developing embryo. Consequently, the four-cell-stage embryos have a very different RNA composition compared with one-cell-stage and two-cell-stage embryos. Neither ribosome occupancy nor RNA expression is positively correlated with protein abundance at the four-cell or the eight-cell stage (Fig. 5g and Extended Data Fig. 8h). Instead, we found that ribosome occupancy and RNA expression at four-cell-stage and eight-cell-stage embryos are much more strongly associated with the protein abundance at the morula stage (Fig. 5g; Spearman rank correlation of 0.66 versus 0.50 at the four-cell stage and 0.69 versus 0.53 at the eight-cell stage; *P* < 2.2 × 10^−16^). These results reveal that the interplay between protein stability and production contribute to the dynamics of protein abundance during mouse preimplantation embryonic development.

## Translation efficiency of transcripts with allelic bias

**Fig. 6:**
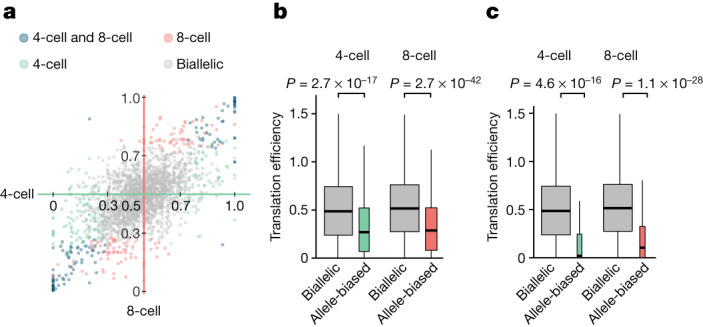
Translation efficiency of transcripts with allele-specific bias in RNA expression. **a**, For each transcript, we aggregated RNA expression across replicates and phased SNPs to determine the ratio of reads supporting the paternal allele to the total. Genes are coloured by allelic bias in RNA expression in four-cell-stage (*x* axis) and eight-cell-stage (*y* axis) embryos. **b**, For the four-cell-stage and eight-cell-stage embryos, translation efficiency (that is, ribosome occupancy divided by RNA expression) of allele-biased and biallelic genes was compared using two-sided Wilcoxon rank sum tests. **c**, Translation efficiency of biallelic genes was compared with those that are monoallelically expressed (paternal ratio of 0 or 1). In the boxplots, the horizontal line corresponds to the median, the box represents the interquartile range and the whiskers extend to 1.5 times the interquartile range.

The parent-of-origin-specific expression is critical for early mammalian embryonic development^45–49^. However, little is known about the translational control of genes that display allele-biased expression. By the four-cell stage, most genes are transcribed from both alleles in a nearly equal ratio (biallelic expression; Fig. 4a). We first identified the set of genes that deviate from this pattern such that one of the alleles accounted for more than 70% of the total transcripts (allele-biased; Fig. 6a). We discovered that genes that display allele-biased expression were significantly less efficiently translated in both four-cell-stage and eight-cell-stage embryos (Fig. 6b; Wilcoxon rank sum test *P* ≤ 2.7 × 10^−17^; median fold change = 0.55). The most extreme form of allelic bias is monoallelic expression. The translation efficiency of such monoallelically expressed genes were even more reduced than biallelic genes (*P* ≤ 4.6 × 10^−16^; median fold change ≤ 0.2, respectively). Furthermore, the observed difference in translation efficiency was consistent regardless of whether a gene is expressed in a paternally or maternally biased manner (Extended Data Fig. 9a,b).

Identification of allele-biased expression from RNA-seq data can suffer from technical biases^50^. To address potential artefacts, we carried out several additional controls. First, we defined a higher confidence set of genes whose allele-biased expression is supported by multiple SNPs. This group had similarly lower translation efficiency (Extended Data Fig. 9c; *P* ≤ 1.4 × 10^−14^; median fold change = 0.57). Next, to rule out potential confounding due to differences in RNA expression or coding sequence length, we selected subsets of biallelic expressed genes such that their RNA abundance and CDS length distribution matched those of allele-biased genes in their respective embryonic stages. The observed difference in translation efficiency remained significant when the matched sets were compared (Extended Data Fig. 9d; *P* ≤ 6.3 × 10^−5^; median fold change ≤ 0.68).

Finally, we tested whether the observed difference in translation efficiency is restricted to the stage of development in which genes have allele-biased expression, or due to intrinsic features that lead to poor translation. When comparing genes with allele-biased expression to those that are biallelically expressed in the four-cell-stage and eight-cell-stage embryos, we found that their translation efficiencies in MII-stage oocytes and the zygote were indistinguishable (Extended Data Fig. 9e–h; *P* values of 0.41 and 0.33). These findings suggest that transcripts were poorly translated specifically in the stage of development in which their RNA expression is allele-biased. Together, our analyses identify a relationship between allele-biased transcription and translation.

## Discussion

Translational control of gene expression has an imperative role in the early stages of mouse embryonic development. However, technological limitations precluded analysis of allele-specific translation regulation due to lack of single-cell and single-embryo resolution. Here we overcame this critical limitation by developing a microfluidic ITP technique named Ribo-ITP. Ribo-ITP isolates RPFs from individual cells via a novel technology in comparison with a recent study that applied single-cell RNA-seq approaches in combination with an RNase digestion step^51^.

Our high-coverage data enabled the characterization of differential translation efficiency and the first analysis of allele-specific ribosome occupancy in mouse preimplantation development. In particular, we discovered that APC/C and several components of the centrosome are translationally upregulated upon fertilization. The zygote relies on maternally deposited mRNAs to initiate a mitotic program by remodelling the cellular environment, transitioning away from meiotic divisions that proceed without centrosomes. Hence, the initial preimplantation mitosis occurs under fundamentally different cellular conditions compared with somatic divisions^30^. Our results revealed translational upregulation of key components involved in this transition^31^.

Single-cell and single-embryo quantitation of ribosome occupancy avoids the heterogeneity contributed by bulk analysis of embryos. This resolution precisely allowed us to detect genes that exhibit allele-specific ribosome engagement. Our analyses suggest differential RBP sites as one possible contributor to allele-specific ribosome occupancy differences. We speculate that preferential use of maternal ribosomes could contribute to the observed differences in translation of maternally and paternally derived RNAs^52^. Future work will differentiate potential parent of origin from sequence-specific differences.

Finally, we assessed the contribution of translation in determining the proteome of mouse preimplantation embryos. We discovered temporal dynamics that eluded previous RNA expression-based analyses. Examples of similar temporal disconnection between the RNA and protein abundance had previously been observed in *Drosophila* and *Xenopus*^53,54^. Our work extends these studies by experimentally determining the contribution of translation in a mammalian system. Specifically, we found that the ribosome occupancy of germinal vesicle-stage oocytes, and not the zygote, is the strongest predictor of zygotic protein abundance. Future efforts that incorporate protein and RNA stability measurements would be required to address the remaining unexplained variation in protein abundance. This study demonstrates the kind of new biological insights that we can expect from the application of Ribo-ITP, which will help to answer fundamental questions in translational control relevant to samples with limited input amounts, including embryonic tissues, cancer stem cells and transient populations.

## Methods

### PDMS chip fabrication

Moulds were 3D-printed by Proto Labs with WaterShed XC 11122 at high resolution (Supplementary Fig. 1). Reusable moulds were assembled by taping 3D-printed molds to glass slides (5″ × 4″; Ted Pella). Sylgard 184 PDMS monomer and curing agent (4019862, Ellsworth Adhesives) were mixed at a 10:1 (w/w) ratio. The mixture was degassed using a desiccator connected to a vacuum pump, poured over the mold and degassed again until there were no air bubbles. The mold was incubated for at least 16 h at 50 °C. Individual PDMS chips were cut along the lines that form the outer rectangle on the design in Extended Data Fig. 1a. The 5-mm diameter elution well and trailing electrolyte and leading electrolyte reservoirs were made with a biopsy punch (Extended Data Fig. 1b). Before the plasma treatment, glass slides (4″ × 3″; Ted Pella) and the feature side of the PDMS slabs were thoroughly cleaned with tape to remove any dust particles. PDMS chips and glass slides were plasma cleaned with a 115 V Expanded Plasma Cleaner (Harrick Plasma) connected to a Dry Scroll Pump (Agilent) for 2 min at high radio frequency level. The plasma-treated surfaces of the glass and PDMS slabs were immediately brought together to form a covalent bond. Bonded chips were heated at 80 °C on a heat block for at least 2 h to enhance bonding.

### PDMS chip preparation for Ribo-ITP experiments

To ensure clean, RNAse-free chips, we pre-treated the channels and reservoirs of the Ribo-ITP chip by sequential treatment with the following solutions: RNaseZap (100% concentrate), nuclease-free water, 1 M NaOH, nuclease-free water, 1 M HCl, nuclease-free water, 10% (w/v) benzophenone in acetone (for 10 min, replenishing channels as needed to avoid bubble accumulation), methanol and 0.1% Triton X-100. The channel was completely dried after final treatment by fully vacuuming out any remaining liquid in the channel. After securing the chips to a ProteinSimple 302/365nm UV Transilluminator with tape, we added 10% polyacrylamide prepolymer mix (Supplementary Table 6) to the size-selection channel through the elution well. Similarly, 5% polyacrylamide prepolymer mix was loaded into the extraction channel through branch channel 2. To catalyse the polymerization of polyacrylamide on chip, we used a photoactivatable azo-initiator, 2,2′-azobis[2-methyl-*N*-(2-hydroxyethyl)propionamide] (VA-086, Wako Chemicals), at 0.5% final concentration in the prepolymer mixes. UV-driven polymerization (wavelength of 365 nm) was performed for 1 min followed by a 30-s break. This on–off UV cycle was repeated two more times for a total UV exposure time of 3 min. UV intensity was measured as approximately 8.9 mW cm^−^^2^ using a G&R Labs Model 200 UV Light meter with a 365-nm probe. To avoid dehydration of the polyacrylamide gels after polymerization, we filled any open channels and reservoirs with storage buffer (Supplementary Table 6) until use. The chips were protected from light and used within 6 h of preparation.

### ITP setup

The prepared ITP chip was placed on a Dark Reader blue light transilluminator (Clare Chemical) and secured with tape. Storage buffer was removed from the channels and reservoirs using a vacuum. Leading electrolyte pluronic solution (LEp) and MOPS trailing electrolyte pluronic solution (TEp) (Supplementary Table 6) were kept on ice throughout the loading procedure. Pipet tips (200 µl) were kept at −20 °C until the time of the experiment to facilitate manipulation of the pluronic-containing LEp and TEp solutions, which solidify within a minute above 4 °C. Of LEp, 80 µl was loaded in leading electrolyte reservoir 3, filling the reservoir to the top as well as the small section of the channel between the elution well and leading electrolyte reservoir 3 (Extended Data Fig. 1b). Leading electrolyte reservoir 2 was filled with 30 µl LEp, ensuring contact with the polyacrylamide gel present in branch channel 2. The elution well was filled with 20 µl of running buffer (RB). Fluorescent marker oligonucleotides containing a 5′ ATTO fluorophore and 3′ ddC blocking modification (Supplementary Table 7) were added to the sample followed by dilution with sample dilution buffer. The mixture was loaded into the lysate channel through leading electrolyte reservoir 1. Finally, leading electrolyte reservoir 1 was filled with 30 µl LEp and 70 µl TEp was added to the trailing electrolyte reservoir. The negative electrode was placed in the trailing electrolyte reservoir and the positive electrode in the leading electrolyte reservoir. Positive and negative electrodes were placed in leading electrolyte reservoir 3 and the trailing electrolyte reservoir, respectively. A constant current of 300 mA with a maximum voltage of 1.1 kV (Keithley 2410 Sourcemeter) was applied to the channel. Once the trailing end of the fluorescent markers entered the 5% polyacrylamide gel, the branch channel electrode—with a lower current output due to a 510 kΩ (Xikon) resistor on a custom circuit board—was manually applied in leading electrolyte reservoir 1 for approximately 10 s. When the leading edge of the shorter fluorescent marker reached the end of the size-selection channel, the current was suspended. The elution reservoir was thoroughly washed twice with 30 µl nuclease-free water and refilled with 10 µl dephosphorylation buffer (Supplementary Table 6). Current was applied again until the longer fluorescent marker began to enter the elution well. Finally, the purified sample with a 10-µl volume was collected from the elution well into a low-bind PCR tube and immediately stored at −80 °C.

### PAGE and conventional extraction of RNA

Control inputs were prepared as a master mix then aliquoted. For gel extraction samples, input RNA was first processed using Qiagen miRNeasy Micro Kit per the manufacturer’s instructions. RNAs were separated by electrophoresis using 15% TBE-urea polyacrylamide gel (EC6885BOX, Invitrogen). Gel slices were excised and crushed using sterile pestles, followed by soaking in gel extraction buffer^57^ (Supplementary Table 6) on dry ice for 30 min. Samples were then incubated overnight at room temperature, gently transferred on a tabletop shaker and protected from light. Residual gel pieces were removed by centrifugation for 1 min at 21,130*g* through a Corning 0.22-μm sterile filter tube. The recovered eluate was precipitated overnight at −20 °C (300 mM sodium acetate (pH 5.2), 5 mM MgCl_2_, 1.5 µl Glycoblue and 75% ethanol). Samples were pelleted by centrifugation at 4 °C for 1 h at 21,130*g*.

### Gel imaging and quantification

To quantify yield, samples were run on a 15% TBE-urea polyacrylamide gel and visualized using the fluorescent marker oligonucleotides or by SYBR gold staining. Specifically, gels were imaged using Typhoon FLA 9500 (GE Healthcare) with a 473-nm excitation wavelength and low pass band filter compatible with ATTO 488 fluorophore and SYBR gold stain. For high-resolution imaging, pixel size was minimized (10–25 µm) and photomultiplier tube (PMT) settings were optimized by using the scanning feature of Typhoon to avoid image oversaturation, typically resulting in a value between 250 and 500 V. The images were analysed using ImageJ software v.1.52 (NIH). The raw integrated density (RID) for background signal (RID_background_) was measured by quantifying average RIDs from representative blank areas. RID_background_ was normalized to account for the ratio of the target (*A*_sample_) to the background area (*A*_background_) such that

$${{\rm{Background}}}_{{\rm{normalized}}}={{\rm{RID}}}_{{\rm{background}}}\times ({A}_{{\rm{sample}}})/({A}_{{\rm{background}}})$$

The normalized background value was subtracted from all samples to quantify normalized sample RID values. The percent yield was defined as the ratio of the normalized RID values to the mean of background-normalized input samples. For display purposes only, the contrast and brightness of some images were adjusted in ImageJ and exported as tiff files for figures. The full scans are displayed in Supplementary Fig. 1.

### Yield comparison between on-chip method and conventional RNA extraction

Input controls and experimental samples were prepared with a final total amount of 40 ng, 20 ng, 2 ng, 400 pg or 40 pg of ZR small RNA ladder (R1090, Zymo Research) including 17, 21, 25 and 29-nt RNA oligonucleotides. Ribo-ITP was performed as described, with a final elution in 12 µl RB. Samples for gel extraction were first processed with the miRNeasy Micro kit (Qiagen), followed by extraction using the crush-and-soak approach^57^. Only the 25-nt and 29-nt bands were extracted. For 40 ng and 20 ng samples, fluorescent marker oligonucleotides were spiked into each sample and a final 15% TBE-urea polyacrylamide gel was run as described above. Only the 25-nt and 29-nt bands were quantified to determine the final yield.

To quantify yield for the ultra-low input samples (2 ng, 400 pg and 40 pg inputs), all experimental and input control samples were brought to 16 µl with nuclease-free water. Subsequently, 2 µl T4 polynucleotide kinase (PNK) buffer, 1 µl T4 PNK (NEB) and 1 µl ATP [γ-32P]-3000 Ci mmol^−^^1^ (10 mCi ml^−^^1^) (NEG002A500UC, Perkin Elmer) were added and incubated for 30 min at 37 °C. After incubation, unincorporated nucleotides were removed with the RNA Clean and Concentrator-5 kit (R1013, Zymo Research) according to the manufacturer’s instructions. RNA was eluted with 14 µl nuclease-free water, mixed with 2× denaturing gel loading dye (Supplementary Table 6) and denatured for 90 s at 80 °C. The samples were electrophoresed, then the gel was incubated in nuclease-free water for 5 min followed by a 30 min incubation in a 30% methanol and 5% glycerol solution. Both incubations were done on a rocking platform at room temperature. After the incubations, the gel was placed between pre-wetted cellophane sheets (1651779, Bio-Rad) and dried for 2 h in a GelAir drying system (Bio-Rad). The dried gel in cellophane was exposed for at least 12 h to a BAS-IP MS phosphor screen (28956475, GE Healthcare). The phosphor screen was imaged with a Typhoon FLA 9500 (GE Healthcare) using 500 V PMT at 50-µm resolution. The image was visualized and quantified using ImageJ software; only the 25-nt band was quantified as described above. All samples were processed in quadruplicate, with the exception of the Ribo-ITP sample with an RNA ladder input of 20 ng (*n* = 3).

### Cell culture

Human K562 cells were grown in RPMI 1640 medium (Gibco) supplemented with 10% fetal bovine serum (Gibco) and 1% penicillin–streptomycin (Gibco) and incubated at 37 °C with 5% CO_2_ to a density of approximately 2.5 × 10^5^ cells per ml. Cells were regularly tested for mycoplasma contamination. The identity of the K562 cell line was authenticated using short tandem repeat profiling from the American Type Culture Collection.

### Size selection of purified RNA

To demonstrate the size-selection capacity of our on-chip approach, we prepared an MNase-digested RNA sample from K562 cells. In brief, 3 µl MNase (M0247S, NEB) was added to a clarified K562 lysate from approximately 5 million cells and digested for 30 min at 37 °C, followed by RNA extraction with the miRNeasy Micro kit (Qiagen) per the manufacturer’s instructions. Ribo-ITP inputs contained 100 ng of the digested, purified RNA. Ribo-ITP was performed as described, with modifications to the collection method. Once the fluorescent marker band reached the interface of the 5% and 10% polyacrylamide gels, the current was suspended and RB was replaced with 12 µl of fresh RB. Ribo-ITP continued until the first fluorescent marker reached the edge of the elution well. The 12 µl of RB in the elution well was collected as fraction 1. The well was washed twice with RB then refilled with 12 µl RB. Current was applied again until the front edge of the trailing fluorescent marker began to enter the elution well, and the 12 µl RB elution was collected as fraction 2. The elution well was refilled with 12 µl RB and Ribo-ITP was continued for 2 min. The final 12 µl elution was collected as fraction 3. Control inputs were prepared with the same amounts of bulk RNA and fluorescent markers, then brought to 12 µl with RB. Gel electrophoresis, imaging and quantification were performed as described.

### Ribosome profiling sample preparation and monosome isolation

Approximately 10 million K562 cells were pelleted, washed twice with PBS and immediately flash-frozen in liquid nitrogen. Cells were lysed in 400 µl cold lysis buffer (Supplementary Table 6) for 10 min on ice and pipetted to homogenize. The lysates were clarified by centrifugation at 1,300*g* for 10 min at 4 °C. Clarified supernatants were digested with 5 µl MNase (M0247S, NEB) and incubated for 30 min at 37 °C. Digestions were stopped with 20 mM ribonucleoside vanadyl complex (S1402S, NEB). The samples were then loaded onto 20–50% sucrose gradients and ultracentrifuged in a SW41 Ti swinging-bucket rotor (331362, Beckman) at 38,000 rpm for 2.5 h at 4 °C. The samples were fractionated using a Biocomp gradient fractionator. RNA was extracted from the monosome fractions with the miRNeasy Micro kit (Qiagen). One-third of the eluate was electrophoresed through a 15% TBE-urea polyacrylamide gel. The ribosome footprints of approximately 17–35 nt were gel extracted using the crush-and-soak method as described. Final sample resuspension after ethanol precipitation was in 18 µl of nuclease-free water. The purified RNA was dephosphorylated with 1 µl of T4 polynucleotide kinase (NEB) in 1× T4 PNK buffer for 1 h at 37 °C. Dephosphorylated ribosome footprints were then ethanol precipitated (300 mM sodium acetate, 2.5 volumes of ethanol and 1.5 µl of GlycoBlue) overnight at −20 °C. Precipitated RNA was eluted in 10 µl nuclease-free water. The RNA was normalized to 350 ng in 6 µl of nuclease-free water before library preparation.

For 100-cell ribosome profiling experiments, K562 cells were pelleted, washed twice with PBS and diluted to 100 cells in 5 µl cold lysis buffer containing cycloheximide. MNase stock (2,000 gel units per microlitre; NEB) was diluted 1:50 and 1 µl of the dilution was added to the samples. Digestion was performed for 30 min at 37 °C in a thermal cycler with a heated lid. EGTA (1 µl) was added to a final concentration of 10 mM to inhibit further digestion. Samples were placed on ice until processing through Ribo-ITP. Three replicates each were prepared for conventional ribosome profiling and Ribo-ITP.

For single-cell Ribo-ITP experiments, cells were pelleted at 300*g* for 5 min. The cells were washed with 1× PBS and resuspended to achieve approximately 1 × 10^6^ cells in 1 ml of PBS containing 0.1% BSA (Sigma) with 1 µg ml^−1^ DAPI. The cells were passed through a strained cap to attain a single-cell suspension and sorted with the Sony MA9000 Cell Sorter or BD FACSAria Fusion Flow Cytometer into Eppendorf LoBind 96-well plates containing 5 µl cold lysis buffer with cycloheximide. Singlet cells were defined by gating on FSC-A/SSC-A, SSA-H/SSC-W, FSC-A/FSC-H and FSC-A/histogram. Live cells were selected using DAPI-negative gating. The plates were sealed and flash frozen in liquid nitrogen immediately after the sort was completed. The lysate was incubated at 37 °C with 1 µl of a 1:150 dilution of MNase stock (2,000 gel units per microlitre; NEB) for 30 min or 1 µl of a 1:300 dilution of RNase I (100 U µl^−1^; Ambion) for 15 min. The MNase digestion was stopped by adding EGTA to a final concentration of 10 mM. Sodium dodecyl sulfate was added to a final concentration of 0.1% to samples digested with RNase I. The lysates were held on ice until processing through Ribo-ITP.

### Mouse oocyte isolation

All experiments using mice by the Mouse Genetic Engineering Facility were approved by the Institutional Animal Care and Use Committee at the University of Texas at Austin (protocol ID: AUP-2022-00114). Mice were housed at 22 °C (range 20–24 °C) under 12 h of light–dark cycles. The humidity was not controlled. Oocytes were collected from superovulated C57BL/6J female mice (approximately 8 weeks old) as previously described^18^. One hour after human chorionic gonadotropin (hCG) injection, the ovaries were placed in a 3-cm dish containing FHM medium (F1114, Cytospring), and germinal vesicle-stage oocytes were released by scraping the surface of the ovaries with #5 Dumont forceps (Roboz). MII-stage oocytes were isolated from the oviducts approximately 14 h after hCG injection. Cumulus cells were removed from the oocytes by treatment with 1 mg ml^−1^ hyaluronidase (H3884, Sigma) in FHM medium. Both germinal vesicle-stage and MII-stage oocytes were rinsed through three drops of FHM medium and then through three drops of 20 mg ml^−1^ BSA (A3311, Sigma) in PBS (SH30028.02, Hyclone). The oocytes were placed individually in 0.2-ml PCR tubes using a finely pulled glass pipette under a stereomicroscope and flash frozen in liquid nitrogen. The liquid volume transferred with the oocytes was less than 0.5 µl. No statistical analyses were used to determine sample size. Given the observational nature of the study, no randomization or blinding was used.

### In vitro fertilization using CAST/EiJ sperm

Sperm was frozen from CAST/EiJ male mice as previously described^58^ and stored in liquid nitrogen. For in vitro fertilization, oocytes were isolated from C57BL/6J female mice approximately 15 h after hCG injection, and in vitro fertilization was performed using thawed CAST/EiJ sperm^59^. One-cell, two-cell, four-cell and eight-cell embryos were collected 21.5, 39, 62 and 69 h after hCG injection, respectively. Fertilized oocytes were cultured overnight to the two-cell stage in a 150 µl drop of HTF medium (mH0113, Cytospring). For development to the four-cell and eight-cell stages, two-cell embryos were cultured in KSOM medium (K0114, Cytospring). Embryos were placed individually into 0.2-ml PCR tubes and flash frozen in liquid nitrogen. All samples were processed with Ribo-ITP within 48 h of collection.

A working lysis buffer solution was prepared by adding 1 µl of the MNase (NEB) (1:50 dilution) per 5 µl lysis buffer. To lyse the mouse samples, 6 µl of working lysis buffer was added directly to the frozen cell-containing droplet. Digestion was immediately performed for 30 min at 37 °C in a thermal cycler with a heated lid. EGTA (1 µl) was added to a final concentration of 10 mM to inhibit further digestion. Samples were placed on ice until processing through Ribo-ITP.

### Ribosome profiling library preparation and sequencing

Conventional ribosome footprint libraries following monosome isolation (that is, 350 ng RNA samples in 6 µl nuclease-free water) were generated using the Clontech SMARTer smRNA-seq kit using eight PCR cycles (Takara Bio). Of the PCR, 30 µl was purified with AMPure XP beads (A63880, Beckman Coulter) according to the manufacturer’s instructions and eluted with 30 µl nuclease-free water. The final size selection was performed with the BluePippin system (Sage Science) using 3% dye-free agarose cassettes (BDQ3010, Sage Science).

For Ribo-ITP experiments with human K562 cells and mouse samples, the D-Plex Small RNA-seq kit (C05030001, Diagenode) with minor modifications was used as detailed below. The dephosphorylation reaction was supplemented with 0.5 µl T4 PNK (NEB) and the reaction was incubated for 25 min. For reverse transcription, the template switching oligo was diluted 1:2 in nuclease-free water. All 100-cell human samples and three of the MII-stage oocytes were processed using the single index module; whereas the other mouse samples were processed using the unique dual index module. Half of the complementary DNA (cDNA) was amplified for 17 PCR cycles and a 1:4 dilution of the resulting library was assessed by the Agilent Bioanalyzer High Sensitivity DNA kit. The concentrations of the target peaks were used to pool samples with approximately equimolar representation. AMPure XP bead cleanup (1.8×) was performed followed by size selection using 3% agarose, dye-free gel cassettes with internal standards (BDQ3010, Sage Science) on the BluePippin platform. Tight parameter settings of the 173–207-bp range were used for samples prepared with the single index module. Tight parameter settings of the 183–217-bp range were used for samples prepared with the unique dual index module. For the RNase I-digested single-cell libraries, final size selection was performed by PAGE purification of 200-bp products. Samples were sequenced on an Illumina NovaSeq 6000. For mouse samples, five, five, five, three, three and four biological replicates were used for germinal vesicle, MII, one-cell, two-cell, four-cell and eight-cell stages, respectively.

### Single-cell and single-embryo RNA-seq

Total RNA-seq libraries were prepared with Smart-seq3 V.3 (ref. ^60^), with modifications. Unfertilized mouse samples (germinal vesicle and MII) and in vitro-fertilized mouse samples (one-cell, two-cell, four-cell and eight-cell stage) were lysed and reverse transcribed as described. cDNA was pre-amplified with 13 PCR cycles and bead purified with AMPure XP (1.8×) with a final elution in 5 µl nuclease-free water. Of pre-amplified cDNA, 1 µl was assessed by the Bioanalyzer High Sensitivity DNA kit to confirm successful pre-amplification and proper size profile. Another 1 µl was assessed on Qubit using the double-stranded DNA high sensitivity (HS) assay to quantify the pre-amplified cDNA. Samples were diluted with nuclease-free water and normalized to 600 pg inputs (100 pg µl^−1^) and subjected to tagmentation and post-tagmentation PCR. The tagmentation and subsequent PCR were scaled up 6×: precisely, 600 pg pre-amplified cDNA was tagmented with 6 µl of tagmentation mix, 9 µl of Nextera Index primers were added and 18 µl of tagmentation PCR mix was used. Sixteen PCR cycles were performed followed by equivolume sample pooling (12 µl of each PCR product) and AMPureXP purification at a 1× ratio. The final library size distribution and concentration were assessed with the HS DNA Bioanalyzer. Sequencing was performed with Nova Seq 6000 with paired-end reads (using 100 cycle kits: 60 + 40). For germinal vesicle, MII, one-cell, two-cell, four-cell and eight-cell stages, four, four, four, four, two and four biological replicates were sequenced, respectively.

### Computational processing of ribosome profiling data

Ribosome profiling data were processed using RiboFlow^61^. We extracted the first 12 nt from the 5′ end of the reads using UMI-tools^62^ version 1.1.1 with the following parameters: “umi_tools extract -p “^(?P<umi_1>.{12})(?P<discard_1>.{4}).+$”–extract-method=regex”. The 4 nt downstream of the unique molecular indexes (UMIs) are discarded as they are incorporated during the reverse transcription step. Conventional ribosome profiling samples did not include UMIs.

Next, we clipped the 3′ adapter AAAAAAAAAACAAAAAAAAAA, from the Ribo-ITP data, using cutadapt^63^ version 1.18 with the parameters “-a AAAAAAAAAACAAAAAAAAAA–overlap=4–trimmed-only”. For conventional ribosome profiling data, we removed the poly(A) tails and the first 3 nt of the reads using “cutadapt -u 3 -a AAAAAAAAAA–overlap=4–trimmed-only”.

After UMI extraction and adapter trimming, reads were aligned to ribosomal and transfer RNAs using Bowtie2 (ref. ^64^) version 2.3.4.3. The unaligned reads were mapped to a manually curated transcriptome. We retained alignments with mapping quality greater than 2 followed by deduplication using UMI-tools when applicable. In deduplication of external libraries without UMIs, a set of reads with the same length that were mapped to an identical nucleotide position were collapsed into a single read. As the last step, .ribo files were created using RiboPy^61^ version 0.0.1. All subsequent analyses used ribosome footprints that were 29–35 nt in length.

For analyses involving nucleotide-resolution data, we determined the A-site offset for each ribosome footprint length using translation stop site metagene plots. Specifically, for each read length, we identified the highest peak upstream of the translation stop site and used the distance to the annotated stop site as the offset.

To assign ribosome footprints to coding reading frames (0, 1 and 2), we first calculated the distance between the 5′ end of the footprint and the first nucleotide of the coding sequence and took modulo 3 of the distance. Next, ribosome footprints were partitioned by their length and the 2 nt upstream and 1 nt downstream of the 3′ end of the footprint. For each group, we determined the total number of reads, assigned to each reading frame, giving us three numbers (*S*_0_, *S*_1_ and *S*_2_) where *S*_*i*_ is the total number of footprints in the frame *i*. We cyclically shifted these numbers so that the maximum number was assigned to the first component. After cyclic shifts, we aggregated all triplets component-wise. The resulting triplet (*T*_0_, *T*_1_ and *T*_2_) provides the adjusted reading frames where *T*_*i*_ is the corrected number of footprints in the frame *i*. We compared the resulting reading frame distribution (*T*_0_, *T*_1_ and *T*_2_) to the randomly distributed frames, where the expected value is (*T*_0_ + *T*_1_ + *T*_2_)/3 for each frame (Chi-squared statistic, *P* < 2.2 × 10^−16^ for all experiments).

### Computational processing of RNA-seq data

The 5′ adapter sequence ‘ATTGCGCAATG’ was clipped from the first read in the pair using cutadapt^63^ version 1.18. Clipped reads shorter than 8 nt were removed using: ‘cutadapt -j 4–trimmed-only -m 8 -g ATTGCGCAATG’. We then extracted the next 8 nt corresponding to the UMIs from the first read in the pair and appended them to the headers (of FASTQ files) of both read pairs using UMI-tools with the following parameters: ‘umi_tools extract–bc-pattern NNNNNNNN’. After UMI extraction, we used the second read in the pair (40 nt) for all subsequent analyses.

After filtering out reads aligning to a reference of rRNAs and tRNAs, the remaining reads were aligned to a transcriptome reference in which SNPs were masked with Ns (see the next section for details); thereafter, we retained only the alignments with mapping quality greater than 2. We then collapsed reads that aligned to the same transcript using their respective UMIs: ‘umi_tools dedup–per-contig–per-gene’. For each transcript, we counted the number of reads aligning to the coding sequence. We used Bowtie2 (ref. ^64^) for all alignments and SAMtools^65^ version 1.11 for processing BAM files.

### Comparison with polysome profiling

The transcripts with validated changes in polysomal association between germinal vesicle-stage and MII-stage oocytes were obtained from Supplementary Figs. 2 and 3 of Chen et al.^18^. Of the 29 genes with quantitative reverse transcription PCR-validated changes in polysomal association, 28 had the reported direction of effect when comparing the mean of the centred log ratio (clr)^66^ across the replicates. Specifically, let *M* be the geometric mean of all the genes with non-zero counts and let *g* be the raw counts for a specific gene. Then, clr of *g* is computed as ${\rm{c}}{\rm{l}}{\rm{r}}(\,g)={\rm{l}}{\rm{n}}\frac{g}{M}$.

### The relationship between RNA expression and poly(A) tail length

Previously, poly(A) tail length in germinal vesicle-stage mouse oocytes was measured using both short-read sequencing (TAIL-seq)^67^ and PacBio sequencing (PAIso-seq)^68^. The processed data were obtained from http://ftp.ebi.ac.uk/pub/databases/microcosm/tailseek/ and https://github.com/niehu2018/GV_oocyte_PAIsoSeqAnalysis/tree/master/results. For each gene, we averaged the poly(A) tail measurements across replicates and transcripts.

To determine the effect of oligo(dT) priming on our RNA expression measurements, we reanalysed the only publicly available data from mouse zygotes that did not use poly(A) selection in its RNA measurements (SUPeR-seq)^69^. We downloaded the gene-level expression data from GSE53386 and calculated the mean fragments per kilobase per million mapped reads across the five replicate experiments from wild-type mouse zygotes. We quantified the difference in RNA expression between these measurements and ours using the log_2_ ratio of the normalized values.

**Extended Data Fig. 10:**
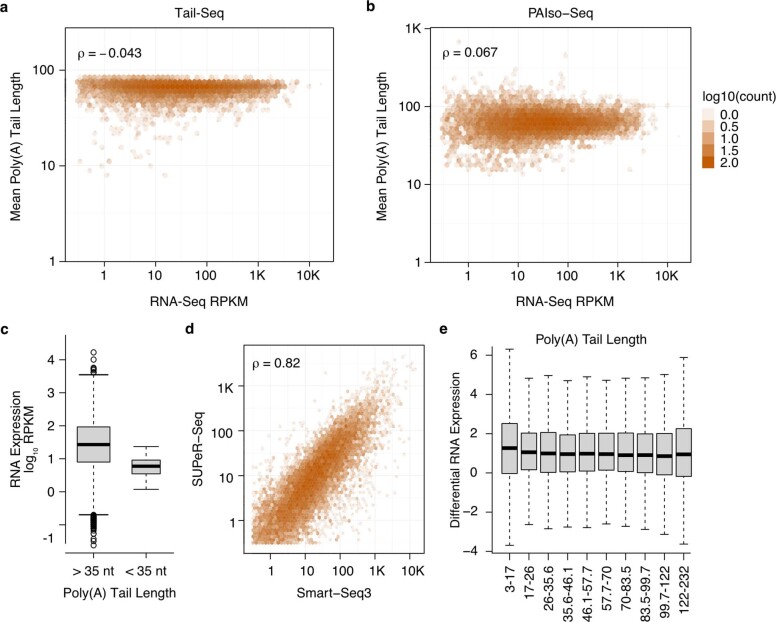
RNA expression measurements using Smart-seq3 do not exhibit a systematic bias as a function of poly(A) tail length. poly(A) tail length of transcripts from mouse GV-stage oocytes were retrieved from two previous studies **a**, Tail-Seq (n = 3)^67^ or **b**, PAIso-seq (n=2)^68^. Spearman rank correlation coefficients between our RNA-Seq measurements and poly(A) tail length was shown in the top left corners highlighting the lack of a strong relationship between the two variables for both Tail-Seq and PAIso-seq. **c**, Transcripts with shortest poly(A) tails (<35nt corresponding to the lowest 1% in TAIL-Seq and the lowest 3.7% in PAIso-seq) were compared to all other transcripts with respect to RNA expression measured by Smart-Seq3. The median of the distribution is shown with the horizontal line. The box depicts the interquartile range, the whiskers extend from the hinge to at most 1.5 times the interquartile range. **d**, Our RNA-seq measurements from zygotes using Smart-seq3 (n = 4), which uses oligo(dT) priming, was compared to measurements using SUPeR-Seq (n = 5)^69^, which provides poly(A) independent quantification. Spearman rank correlation between the two approaches is shown (p-value < 2.2 × 10^−16^). **e**, The log_2_ ratio of SUPeR-Seq to Smart-seq3 expression values (y-axis) were calculated for each gene and grouped by their respective poly(A) tail lengths (x-axis) as previously reported^41^. While there is not a systematic bias in expression measurements as a function of poly(A) tail length, transcripts with the shortest tails (<17nt) were slightly underestimated in Smart-seq3 (Kruskal-Wallis rank sum test; p-value 0.016).

We found a very weak correlation between measured poly(A) tail length and our RNA expression measurements from germinal vesicle-stage oocytes (Spearman correlation of −0.04 for Tail-seq and 0.07 for PAIso-seq; Extended Data Fig. 10a,b). To rule out the possibility that the observed weak correlation may be due to poor reliability of the poly(A) tail measurements, we compared PAIso-seq and Tail-seq measurements and found that they were moderately correlated with each other for the set of transcripts that were measured more than once (Spearman correlation coefficient of 0.42; *P* < 2.2 × 10^−16^).

Even though poly(A) tail length does not systematically confound measured RNA expression, the abundance of transcripts with extremely short poly(A) tails can still be underestimated. Indeed, the subset of genes with the shortest average poly(A) tail length (less than 35 nt corresponding to the lowest 1% in TAIL-seq and the lowest 3.7% in PAIso-seq) had significantly lower RNA expression measurements (Extended Data Fig. 10c; Wilcoxon rank sum test *P* < 2.2 × 10^−16^; Extended Data Fig. 10c).

The observed lower expression of transcripts with the shortest poly(A) tails could stem from a technical artefact of using poly(A) selection. Alternatively, mRNAs with the shortest poly(A) tails may have intrinsically lower expression. To differentiate these two alternatives, we compared our measurements with SUPeR-seq, a method that does not rely on poly(A) selection^69^. As expected, SUPeR-seq measurements were highly correlated with our own measurements (Spearman correlation coefficient of 0.82; *P* < 2.2 × 10^−16^; Extended Data Fig. 10d). More importantly, when comparing the difference in measured RNA abundance between the two methods, there was only a minimal association as a function of poly(A) tail length (Extended Data Fig. 10e; Kruskal–Wallis rank sum test; *P* value of 0.016).

Together, these results suggest that there is not a systematic bias in Smart-seq3-based RNA expression measurements as a function of poly(A) tail length. However, the expression of genes with the shortest poly(A) may be slightly underestimated.

### The relationship between translation efficiency and poly(A) tail length

Mouse embryos from early one-cell, two-cell and eight-cell stages were previously used to determine poly(A) tail length using an improved version of TAIL-seq (10.5281/zenodo.2640028)^41^. This HDF5 file contained tag counts aggregated by poly(A) tail lengths. We calculated the mean poly(A) tail length of each gene using the instructions by the authors and rhdf5 package version 2.42.0 (https://github.com/grimbough/rhdf5).

For each gene and embryonic stage, we first calculated the density of ribosome footprints and RNA-seq reads across the coding region. These values were then normalized using the centred log ratio^66^ and were averaged across replicates. Translation efficiency of a gene in a given embryonic stage was defined as the ratio of the normalized ribosome occupancy to RNA expression. The bootstrap confidence interval for translation efficiency was calculated by sampling with the replacement of the replicate Ribo-ITP and RNA-seq experiments and repeating the described calculation.

### Allele-specific ribosome occupancy and RNA expression analysis

Given that mouse embryos were obtained by crossing the strains C57BL/6J (maternal) and CAST/EiJ (paternal), we used known strain-specific SNPs to determine the parental origin of the RNA molecules. This allowed us to determine whether the ribosome occupancy or RNA expression of a gene exhibits a maternal or paternal (that is, allele-specific) bias as detailed below.

A list of strain-specific SNPs was obtained in VCF format from https://github.com/sandberg-lab/Smart-seq3/blob/master/allele_level_expression/CAST.SNPs.validated.vcf.gz^60^. We extracted 210,004 distinct SNPs that overlapped with transcript annotations. To avoid alignment biases, we modified our transcriptome reference sequences by masking SNP positions with Ns. Mouse sequencing data were aligned to this masked transcriptome reference.

For allele-specific analyses, we considered the 85,339 SNPs within the coding sequences of transcripts. Given that transcripts in oocytes should solely contain maternal SNPs, we used the data from the MII-stage oocytes to construct a simple error correction model. Specifically, 2.67% and 0.40% of reads contained non-maternal sequences in ribosome profiling and RNA-seq experiments, respectively. These values were used as estimates of the sequencing error percentage (error).

We define the paternal ratio as

$$\frac{{\rm{Number}}\;{\rm{of}}\;{\rm{reads}}\;{\rm{from}}\;{\rm{paternal}}\;{\rm{alleles}}+1}{{\rm{Number}}\;{\rm{of}}\;{\rm{reads}}\;{\rm{from}}\;{\rm{paternal}}\;{\rm{alleles}}+1+{\rm{number}}\;{\rm{of}}\;{\rm{reads}}\;{\rm{from}}\;{\rm{maternal}}\;{\rm{alleles}}+1}.$$

For one-cell to eight-cell embryos, we then calculated the error-corrected paternal ratio, ${{\rm{paternal}}}_{{\rm{corrected}}}$ as:

$${{\rm{paternal}}}_{{\rm{corrected}}}=\frac{300\times {{\rm{paternal}}}_{{\rm{observed}}}-{\rm{error}}\times 100}{300-4\times {\rm{error}}}$$

where, ${{\rm{paternal}}}_{\mathrm{observed}}$ is the uncorrected percentage. We derived this equation from the below model under the assumption that sequencing errors are random:

$${{\rm{paternal}}}_{{\rm{observed}}}={{\rm{paternal}}}_{{\rm{corrected}}}\times \frac{100-{\rm{error}}}{100}+(100-{{\rm{paternal}}}_{{\rm{corrected}}})\times \frac{100-{\rm{error}}}{3\times 100}.$$

For each embryonic stage, to identify the transcripts whose paternal ratios are significantly different in ribosome profiling compared with RNA-seq, we first aggregated all SNP-containing reads for each transcript across replicates. We retained transcripts with more than ten reads in both ribosome profiling and RNA-seq experiments including at least three maternal and paternal reads. We used a two-sample test for the equality of proportions with continuity correction (prop.test in R; see chapter 3 of ref. ^70^ for details).

Transcripts with 95% confidence intervals of difference in paternal ratios (derived from the test for the equality of proportions), overlapping with the interval (−0.05, 0.05), were filtered out. After adjusting the *P* values using the false discovery rate method, we retained the transcripts with adjusted *P* < 0.2. We further removed transcripts with paternal reads in the MII stage as these probably indicate positions that are prone to alignment errors. As the final step, we applied bootstrapping to establish robustness of the conclusions. Specifically, we randomly sampled replicates with replacement and repeated the statistical testing procedure described above. Twenty-four transcripts with a false discovery rate less than 0.2 in at least 66 out of 100 bootstrap samples were deemed as having differential allelic ratios. There were a total of 187 coding region SNPs differentiating the two alleles among this set of 24 of these transcripts. A list of these SNPs is provided in Supplementary Table 6.

For the analyses described in Fig. 6 and Extended Data Fig. 9, we considered RNA expression data from four-cell and eight-cell embryonic stages. We discarded the genes with fewer than ten parent-of-origin differentiating reads across all replicates of the given embryonic stage. To define genes that display allele-specific bias in expression, we used a bootstrapping approach. For each sample, we randomly selected two or four replicates with replacement from the four-cell and eight-cell RNA expression data, respectively. Reads were then combined across replicates and SNP positions. A gene was deemed paternally or maternally biased if the ratio of the paternal-to-maternal allele supporting reads were greater than 70% in at least 800 out of 1,000 bootstrap samples. The remaining genes were considered as biallelic (or unbiased). In total, we identified 2,239 and 3,707 biallelic, 191 and 334 maternally biased and 103 and 253 paternally biased genes in four-cell and eight-cell stages, respectively. Furthermore, 37 and 47 genes had expression from only one of the alleles in the four-cell and eight-cell stages, respectively. This subset of allele-specific genes was defined as monoallelic. Finally, to define a more high-confidence set of allele-biased genes, we required support from multiple SNPs such that the ratio of SNPs with $\frac{{\rm{Number}}\;{\rm{of}}\;{\rm{reads}}\;{\rm{from}}\;{\rm{paternal}}\;{\rm{alleles}}+1}{{\rm{Number}}\;{\rm{of}}\;{\rm{reads}}\;{\rm{from}}\;{\rm{paternal}}\;{\rm{alleles}}+1+{\rm{number}}\;{\rm{of}}\;{\rm{reads}}\;{\rm{from}}\;{\rm{maternal}}\;{\rm{alleles}}+1}$ > 0.5 was at least 60%. We found that 195 and 323 genes were supported by multiple SNPs in four-cell and eight-cell stages, respectively. To compare the translation efficiency distribution of different gene groups, we used the non-parametric Wilcoxon rank sum test. To estimate the magnitude of the effect size, we report fold changes defined as the ratio of the median translation efficiency of the allele-specific genes to that of biallelic genes.

### Differential expression and translation efficiency analysis

Reads that aligned to coding regions were extracted for all experiments. To determine transcripts with the highest variability in ribosome occupancy across developmental stages, a variance-stabilizing transformation (VST), as described in ref. ^71^, was applied to centred log ratio of ribosome occupancies (‘FindVariableFeatures’ function with the selection method ‘vst’ in the Seurat package v4 (ref. ^72^)). Using the threshold ‘vst.variance.standardized’ > 4.8, we obtained 50 genes.

For every pair of consecutive developmental stages, differential RNA expression and translation efficiency was determined using DESeq2 (ref. ^73^). For calculation of differential translation efficiency, we used the interaction term between the developmental stage and the measurement modality (ribosome profiling or RNA-seq). Default parameters were used for read count normalization and estimation of gene-specific dispersion. Effect size moderation was carried out using the approximate posterior estimation for a generalized linear model^74^. The adjusted *P* value cut-off was set to 0.01 to determine a set of transcripts with significant changes in RNA expression and translation efficiency. Gene set enrichment analyses for gene ontology terms were carried out using FuncAssociate (http://llama.mshri.on.ca/funcassociate/) with default settings^75^.

### Proteomics data and comparison with RNA-seq and ribosome profiling

Tandem Mass Tag-labelling-based proteomics abundance data for one-cell to morula-stage embryos were obtained from Gao et al.^6^. Measurements in all three modalities were available for 3,287 proteins and were used in further analysis. Ribosome occupancy and RNA expression were converted to read density by dividing the read counts by the length of the coding region of each transcript. These values were normalized using a centred log-ratio transformation as implemented in Seurat v4 (ref. ^72^). The translation efficiency was defined as described above. The similarity between RNA expression, ribosome occupancy, translation efficiency and protein abundance was measured using rank correlation with Spearman’s correction^76,77^. The measurement reliability for each modality was estimated using replicate to replicate correlation coefficients (0.53 for translation efficiency, 0.71 for ribosome profiling, 0.79 for RNA-seq and 0.8 for mass spectrometry^6^).

### Weighted transcript region length distribution

For the transcript regions, 5′ UTR, CDS and 3′ UTR, the distribution of weighted region lengths was calculated as follows. First, for each transcript, we determined the ratios of region lengths to the transcript length. Next, we multiplied these ratios with the number of ribosome occupancies in the transcript, giving us weighted ratios of the regions. Then, for each region, we calculated the sum of their weighted ratios across transcripts. Finally, let *w*_5′ UTR_, *w*_CDS_ and *w*_3′ UTR_ be the weighted (*w*) sums of the regions 5′ UTR, CDS and 3′ UTR, respectively. For a region *r*, the weighted length percentage of *r* is $\frac{{w}_{r}}{{w}_{{5}^{{\prime} }{\rm{UTR}}}+{w}_{{\rm{CDS}}}+{w}_{{3}^{{\prime} }{\rm{UTR}}}}\times 100.$

### Characterization of SNP effects on allele-specific ribosome occupancy

All SNPs differentiating the paternal allele from the maternal allele were extracted for the set of transcripts with evidence of differential allele-specific ribosome occupancy (Fig. 4c). These were annotated by their position within the transcript (5′ UTR, CDS and 3′ UTR) and various functional classes as detailed below using bedtools version v2.29.2 (ref. ^78^) with the following options: ‘bedtools intersect -wa -wb’.

SNPs within 5′ UTRs were annotated as candidates for generating translation initiation sequences by matching a 9-nt sequence centred around the SNP to the regular expression ‘[ATCG]+[AG]{1}[ATCG]{2}[ACG]{1}TG[AG]{1}[ATCG]+’ with R base::grepl. SNPs in which either the maternal or paternal allele matched the regular expression were selected. A mouse PD-31 FACS-seq dataset reporting efficiency of non-canonical translation initiation sequences for −4 to +4 (ref. ^79^) was used to score the efficiency of candidate 5′ UTR initiation sites.

### Analyses of RBP motifs

Enrichment or depletion of RBP heptamer motifs was determined by the Transite v1.16.0 *k*-mer transcript set motif analysis method^55^ (Benjamini–Hochberg *P* < 0.0001; Supplementary Table 5) and annotated with oRNAment^56^ RBPs with consensus sequences matching the heptamer. RBP synonyms in the oRNAment database were replaced with their standard gene names (DAZ3: *DAZL*; SF2: *SRSF1*; B52: *SRSF6*; Fusip1: *SRSF10*; and ZNF326: *ZFP326*).

SNPs were intersected with the BED file of oRNAment motifs using bedtools^78^ v2.29.2 after subtracting one from the BED start coordinate to ensure that sequences had the same length as the oRNAment position weight matrices. We filtered 102 out of 1,403 SNPs that intersect RBP motifs due to multiple SNPs being in close proximity (less than 5 nt). The maternal and paternal sequences were scored using the oRNAment matrix similarity score in R v4.0.4. For RBPs with more than one position weight matrix, the maximum of the absolute difference in scores was computed to identify the consensus motif most impacted by a given SNP. Then, RBPs sharing the same consensus motif and overlapping the same SNP were collapsed into a single annotation by computing the median difference in score. Robust standardization of the median difference in score was performed (centre to median, divided by the interquartile range). SNPs predicted to alter RBP binding were selected using the 95th percentile of the absolute, standardized score difference. Finally, RBPs were discarded if they had no mouse homologue or had no detectable expression during the stages of development analysed (A1CF, BOLL, ELAVL4, MSI1, KHDRBS3, PABPC5, RBM4B, TIA1, EIF4G2, RBFOX3, BRUNOL6, RBM23 and SRSF8).

### Reporting summary

Further information on research design is available in the Nature Portfolio Reporting Summary linked to this article.

## Online content

Any methods, additional references, Nature Portfolio reporting summaries, source data, extended data, supplementary information, acknowledgements, peer review information; details of author contributions and competing interests; and statements of data and code availability are available at 10.1038/s41586-023-06228-9.

## Supplementary information

Supplementary Figure 1 This file contains complete gel images for data presented in Fig. 2 and Extended Data Fig. 1.

Reporting Summary

Supplementary Table 1 Basic alignment and filtering statistics for sequencing libraries.

Supplementary Table 2 This table contains the list of SNP positions, their allelic variants and the corresponding amino acids for genes highlighted in Fig. 4.

Supplementary Table 3 This table provides the log-fold changes and adjusted p-values (Benjamini & Hochberg method) corresponding to the differential analysis of TE and RNA expression between developmental stages.

Supplementary Table 4 A background set consisting of detected genes was provided to FuncAssociate^75^ to determine enrichment of gene ontology terms among genes with significant changes in TE. Adjusted p-values were calculated using the permutation based method implemented in FuncAssociate^75^.

Supplementary Table 5 List of enriched and depleted heptamers among genes with differential translation efficiency determined by Transite^55^ using default parameters for the k-mer TSMA method. Adjusted p-values were calculated using the Benjamini-Hochberg method. Heptamers are annotated as existing in the oRNAment database^56^ if the heptamer is within a Hamming distance of 1 with an oRNAment consensus motif.

Supplementary Table 6 This table contains the composition of buffers used in the Ribo-ITP method.

Supplementary Table 7 This table contains the sequences of RNA and fluorescent DNA markers used in the Ribo-ITP method.

Supplementary Video 1 **Representative video of the Ribo-ITP process** Ribo-ITP process using lysis buffer and 1 µl of each 19nt and 36nt fl ddC DNA markers [5 µM].

## Data Availability

Sequencing files for ribosome profiling and RNA-seq experiments are available at the Gene Expression Omnibus (accession number: GSE185732). The oRNAment database files were downloaded from http://rnabiology.ircm.qc.ca/oRNAment (unspecified version, downloaded on 2 December 2021) for RBPs in the *Mus musculus* transcriptome. The following public datasets were used in this study: GSE53386, GSE78634 and GSE162060. Previously generated poly(A) tail length measurements were downloaded from http://ftp.ebi.ac.uk/pub/databases/microcosm/tailseek/, https://github.com/niehu2018/GV_oocyte_PAIsoSeqAnalysis/tree/master/results and 10.5281/zenodo.2640028. A list of strain-specific SNPs was obtained in VCF format from https://github.com/sandberg-lab/Smart-seq3/blob/master/allele_level_expression/CAST.SNPs.validated.vcf.gz.

## Code availability

The code used in the study is available at https://github.com/CenikLab/ribo-itp_paper.
