# Supplementary material for: Single-cell quantification of ribosome occupancy in early mouse development
Source: Nature. 2023 Jun 21;618(7967):1057–64. doi: 10.1038/s41586-023-06228-9 (PMC10307641; doi:10.1038/s41586-023-06228-9)

Fig 2A: Gel images highlighting inputs, RNAs recovered by Ribo-ITP, and gel electrophoresis (G) are shown. Four RNAs of 17, 21, 25, and 29 nt used in the experiment were radioactively labeled at their 5’end. Percent yield was calculated for the 25 nt RNA.

40pg Input


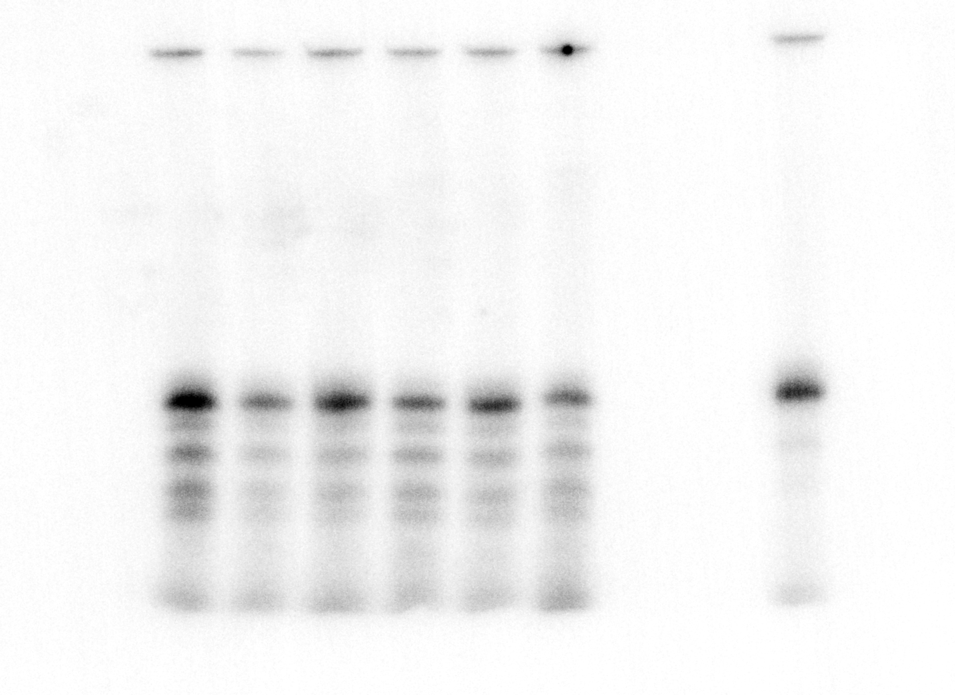


400pg Input


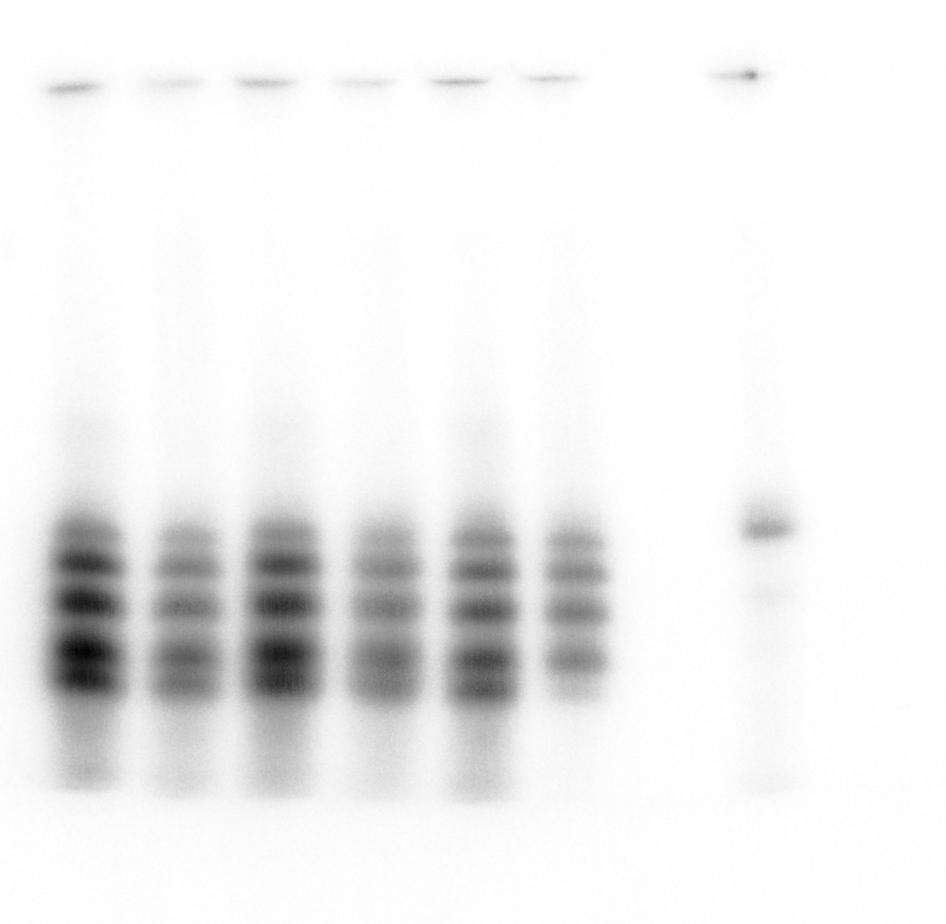


2ng Input


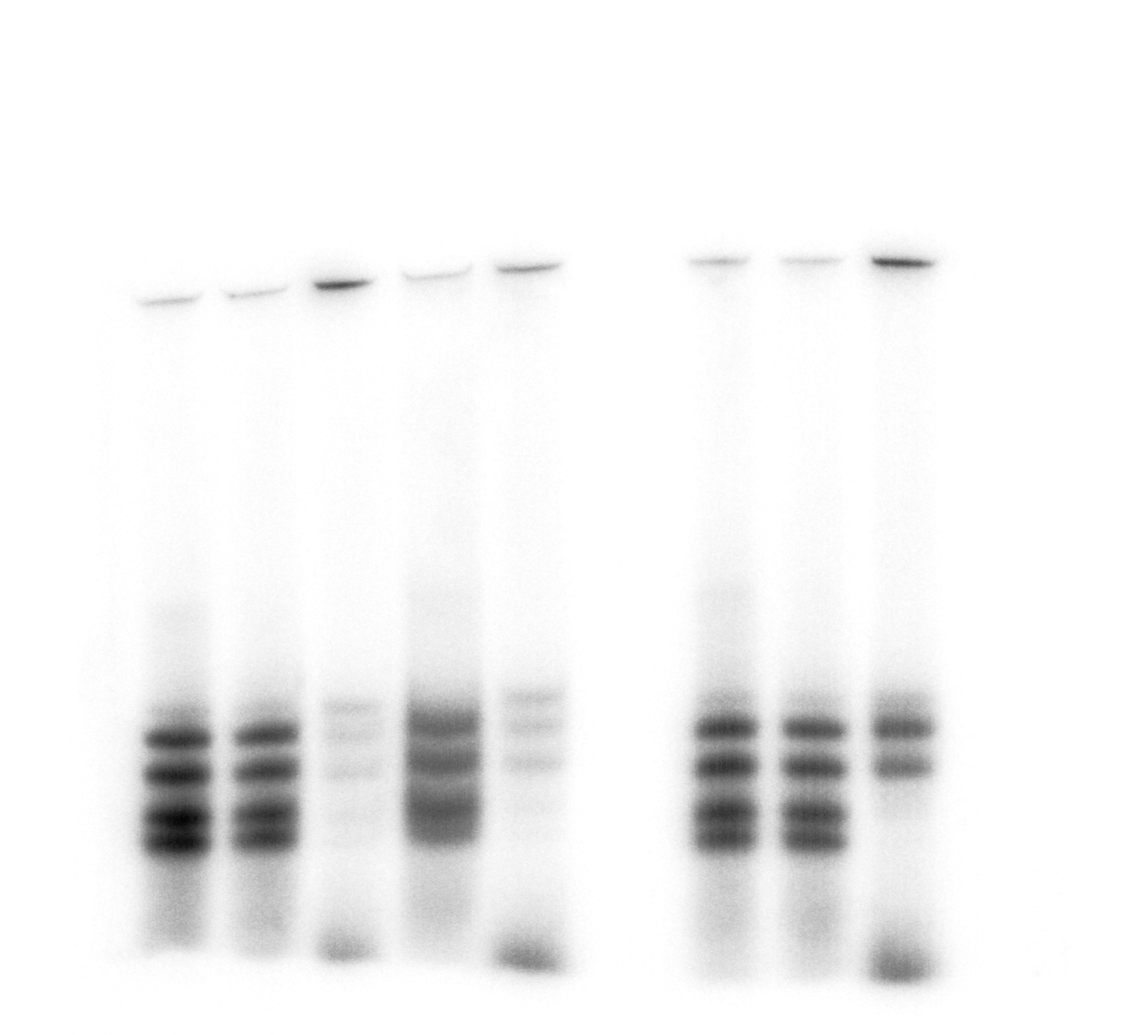


Fig 2B:

| Lane 1: RNA Marker (29, 25, 21, 17 nt) |
| --- |
| Lane 2: Fluorescent nucleotide markers |
| Lane 3: 100 ng of MNase-digested RNA from K562 cells was used as an input for Ribo-ITP after the addition of the two fluorescent marker oligonucleotides |
| Lane 4: Same as Lane 3 without the fluorescent markers |
| Lane 5: RNAs that eluted before the arrival of the shorter fluorescent marker (Fraction 1) |
| Lane 6: The sample flanked by the two fluorescent nucleotide markers (Fraction 2). |
| Lane 7: RNAs that were located behind the longer fluorescent marker (Fraction 3), which typically remain in the channel. |
| Lane 8: RNAs that eluted before the arrival of the shorter fluorescent marker (Fraction 1) |
| Lane 9: The sample flanked by the two fluorescent nucleotide markers (Fraction 2). |
| Lane 10: RNAs that were located behind the longer fluorescent marker (Fraction 3), which typically remain in the channel. |


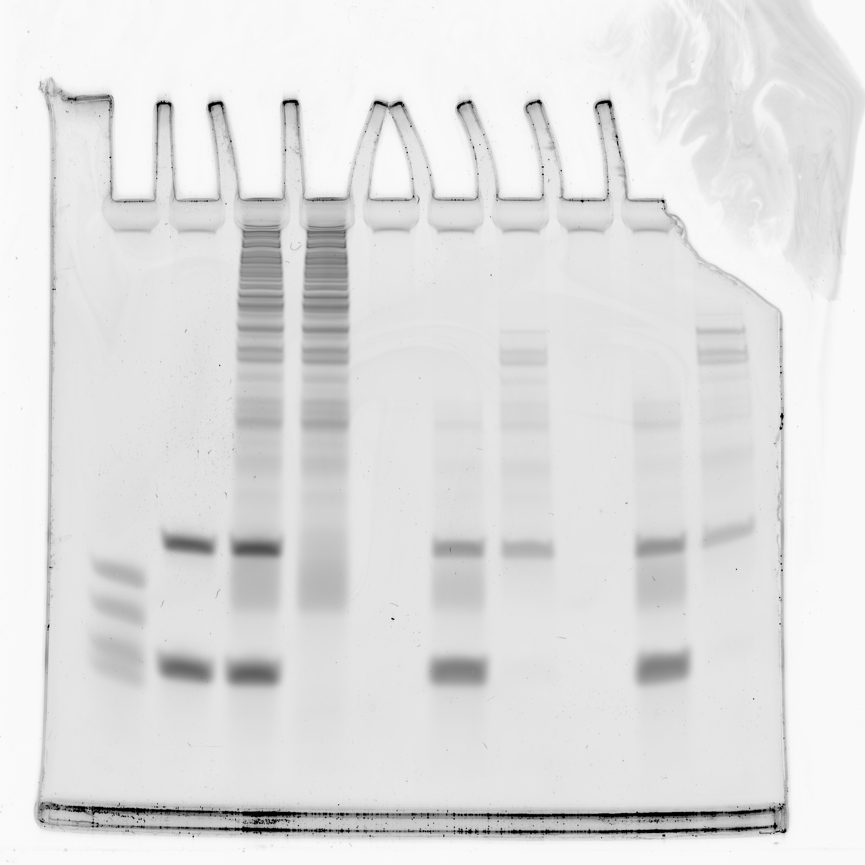


Extended Data Fig 1C: Last three lanes of the gel image displays the relative mobilities of the Zymo Research R1090 small RNA ladder, fluorescent markers used in Ribo-ITP along with and synthetic RNA oligonucleotides.


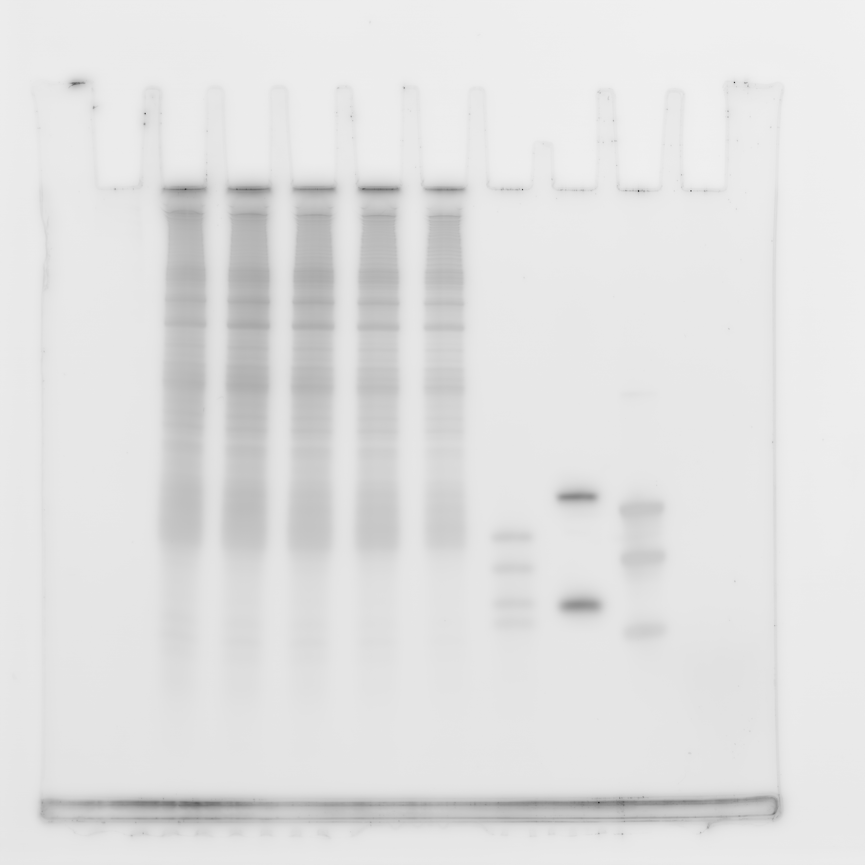


Extended Data Fig 1E: Gel images of control inputs, Ribo-ITP elutions, and gel extraction samples. Four RNA species (17, 21, 25, and 29 nt) were used with total inputs of 20 and 40 ng. Fluorescent marker oligonucleotides were spiked into control and gel extraction samples prior to gel visualization


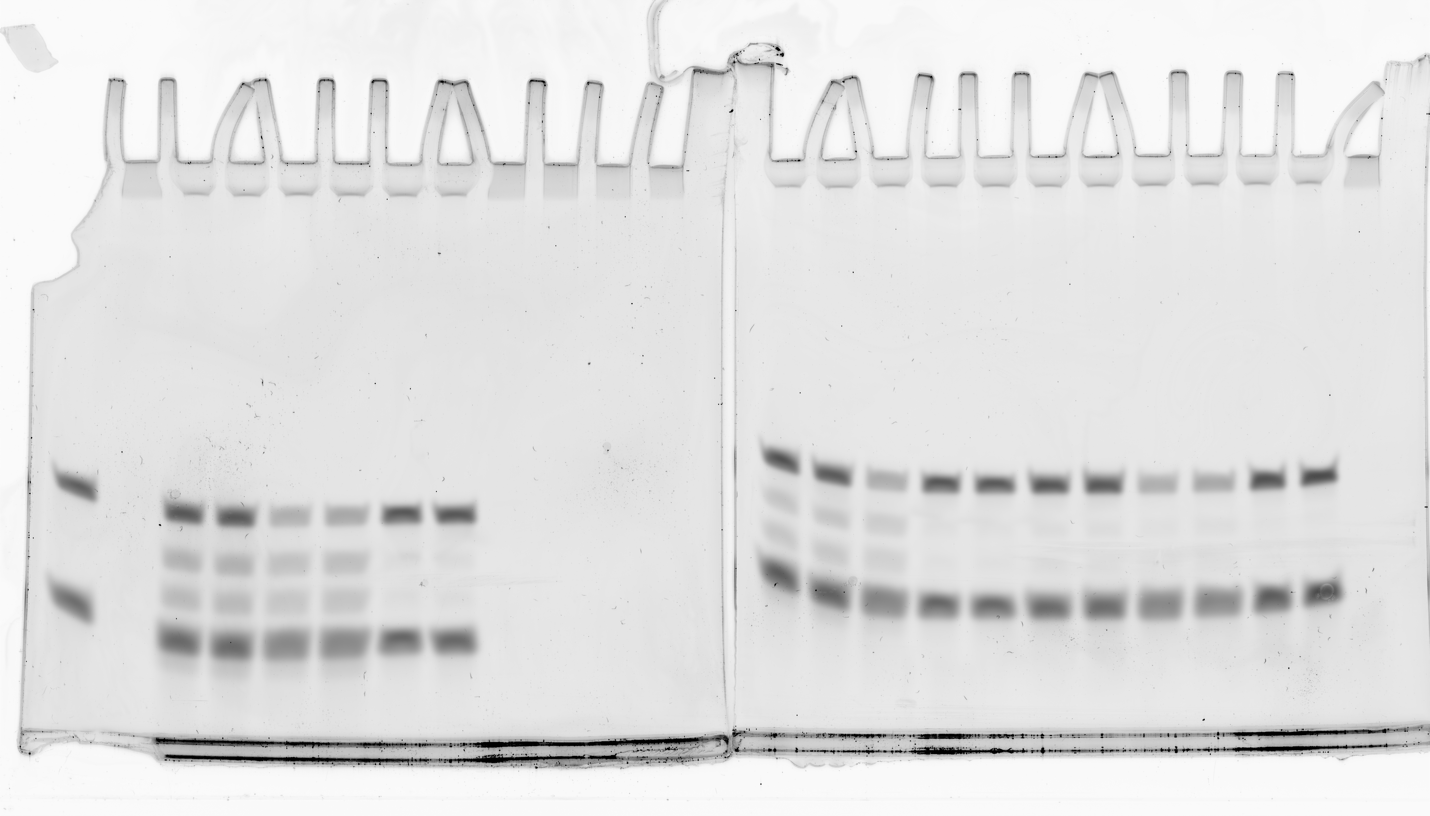


Extended Data Fig 1G: Inputs were prepared by adding 40 ng of RNA to lysates from ~1,000 K562 cells. The RNA consisted of four species ranging from 17 to 29 nt in length. Fluorescent marker DNAs were added to Ribo-ITP samples in addition to EGTA (10 mM; Lane 9). RNA extraction and isolation was done with Ribo-ITP followed by visualization using gel electrophoresis.


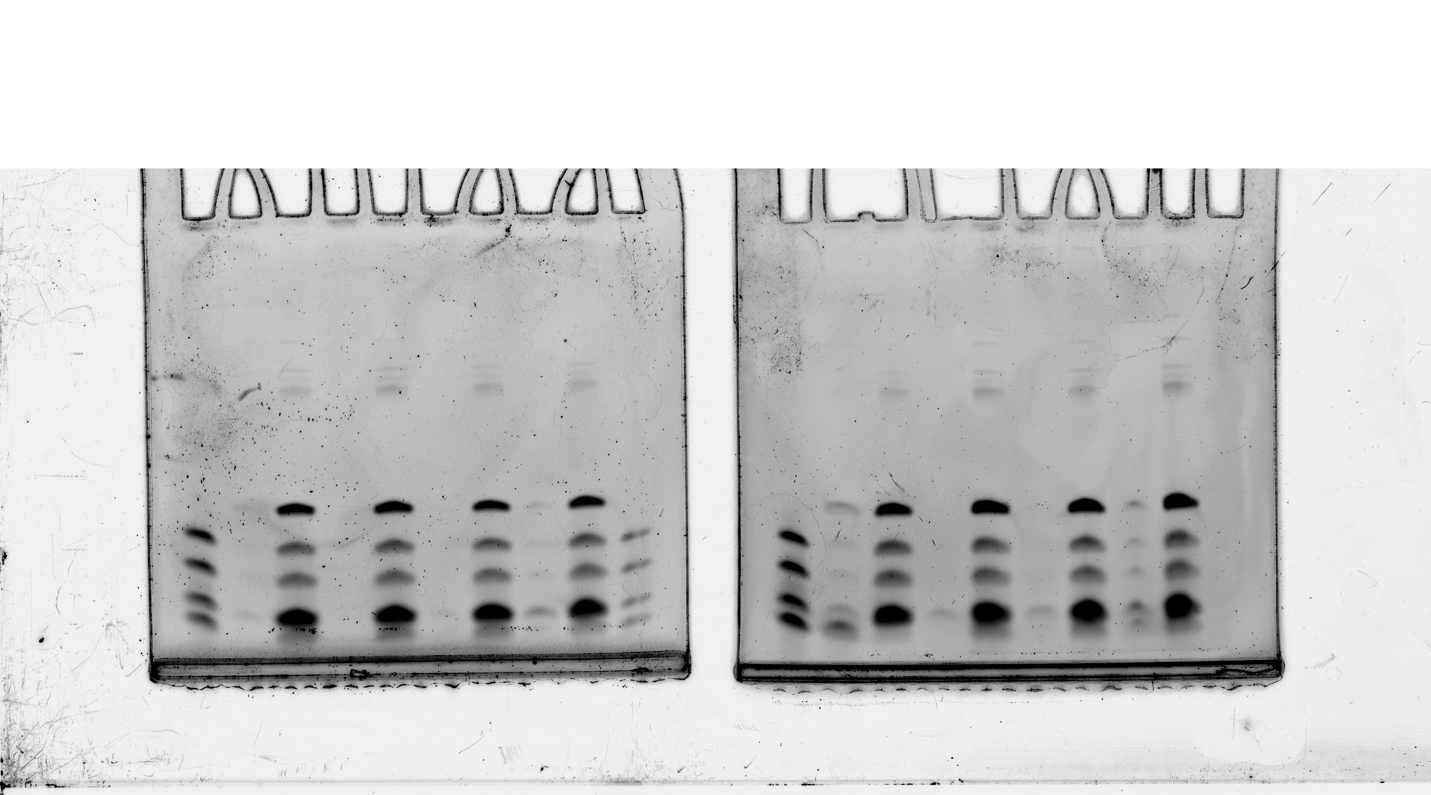

Supplement: Supplementary file 1 — This file contains complete gel images for data presented in Fig. 2 and Extended Data Fig. 1. [file 41586_2023_6228_MOESM1_ESM.docx]
