# Supplementary material for: Single-cell quantification of ribosome occupancy in early mouse development
Source: Nature. 2023 Jun 21;618(7967):1057–64. doi: 10.1038/s41586-023-06228-9 (PMC10307641; doi:10.1038/s41586-023-06228-9)
Supplement: Supplementary file 2 — Reporting Summary [file 41586_2023_6228_MOESM2_ESM.pdf]

nature portfolio

Corresponding author(s): Can Cenik

Last updated by author(s): 04/17/2023

Reporting Summary

Nature Portfolio wishes to improve the reproducibility of the work that we publish. This form provides structure for consistency and transparency in reporting. For further information on Nature Portfolio policies, see our Editorial Policies and the Editorial Policy Checklist.

Statistics

For all statistical analyses, confirm that the following items are present in the figure legend, table legend, main text, or Methods section.

n/a	Confirmed	
☐	☒	The exact sample size (*n*) for each experimental group/condition, given as a discrete number and unit of measurement
☐	☒	A statement on whether measurements were taken from distinct samples or whether the same sample was measured repeatedly
☐	☒	The statistical test(s) used AND whether they are one- or two-sided<br>*Only common tests should be described solely by name; describe more complex techniques in the Methods section.*
☒	☐	A description of all covariates tested
☐	☒	A description of any assumptions or corrections, such as tests of normality and adjustment for multiple comparisons
☐	☒	A full description of the statistical parameters including central tendency (e.g. means) or other basic estimates (e.g. regression coefficient) AND variation (e.g. standard deviation) or associated estimates of uncertainty (e.g. confidence intervals)
☐	☒	For null hypothesis testing, the test statistic (e.g. *F*, *t*, *r*) with confidence intervals, effect sizes, degrees of freedom and *P* value noted<br>*Give P values as exact values whenever suitable.*
☒	☐	For Bayesian analysis, information on the choice of priors and Markov chain Monte Carlo settings
☒	☐	For hierarchical and complex designs, identification of the appropriate level for tests and full reporting of outcomes
☐	☒	Estimates of effect sizes (e.g. Cohen's *d*, Pearson's *r*), indicating how they were calculated

Our web collection on statistics for biologists contains articles on many of the points above.

Software and code

Policy information about availability of computer code

Data collection	Sequencing experiments were done using Illumina NovaSeq 6000.
Data analysis	Data were analyzed using a combination of publicly available and custom software.<br>All custom code developed for this study is available at https://github.com/CenikLab/ribo-itp_paper<br>We provided the analysis parameters of third-party software in the Methods section of our manuscript. The following software versions were used: R: v. 4.0.4 and v. 4.2.1, RiboPy: v. 0.0.1, RiboR: v. 1.3.0, RiboFlow: v. 0.0.0, cutadapt v. 1.18, Bowtie2 v. 2.3.4.3, samtools v. 1.11, rhdf5 v. 2.42.0, Seurat package v4, bedtools v. 2.29.2, Transite v. 1.16.0, ImageJ v.1.52

For manuscripts utilizing custom algorithms or software that are central to the research but not yet described in published literature, software must be made available to editors and reviewers. We strongly encourage code deposition in a community repository (e.g. GitHub). See the Nature Portfolio guidelines for submitting code & software for further information.

Data

Policy information about availability of data

All manuscripts must include a data availability statement. This statement should provide the following information, where applicable:

- Accession codes, unique identifiers, or web links for publicly available datasets
- A description of any restrictions on data availability
- For clinical datasets or third party data, please ensure that the statement adheres to our policy

Sequencing files for ribosome profiling and RNA-seq experiments, together with additional supplemental files, are available at GEO (accession number: GSE185732). The oRNAment database files were downloaded from http://rnabiology.ircm.qc.ca/oRNAment (unspecified version, downloaded December 2, 2021) for RNA-binding proteins in the Mus musculus transcriptome.

The following public datasets were used in this study: GSE53386, GSE78634, GSE162060. Previously generated poly(A) tail length measurements were downloaded from ftp://ftp.ebi.ac.uk/pub/databases/microcosm/tailseek/ , https://github.com/niehu2018/GV_oocyte_PAIsoSeqAnalysis/tree/master/results and doi:10.5281/zenodo.2640028. A list of strain-specific SNPs was obtained in VCF format from https://github.com/sandberg-lab/Smart-seq3/blob/master/allele_level_expression/CAST.SNPs.validated.vcf.gz.

Field-specific reporting

Please select the one below that is the best fit for your research. If you are not sure, read the appropriate sections before making your selection.

☒ Life sciences ☐ Behavioural & social sciences ☐ Ecological, evolutionary & environmental sciences

For a reference copy of the document with all sections, see nature.com/documents/nr-reporting-summary-flat.pdf

Life sciences study design

All studies must disclose on these points even when the disclosure is negative.

Sample size

No statistical methods were used to predetermine sample size. The number of replicates were chosen to assess the reproducibility of the measurements.

Data exclusions

Ribo-ITP libraries were excluded from analysis if the percentage of reads mapping to CDS was less than 60%. This criteria was pre-established based on our experience with previous ribosome profiling experiments. Among human Ribo-ITP experiments, no libraries were removed for 100-cell and 10 million cell experiments. For single cell experiments one out of six libraries was excluded.

Initial optimizations of Ribo-ITP experiments for mouse samples were conducted using oocytes. Ribo-ITP libraries from one GV-stage and nine MII-stage oocytes were excluded. All attempts were successful for single embryo Ribo-ITP experiments from zygotes and 8-cell stage. Four libraries from 2-cell and one from 4-cell stages were excluded.

Replication

In mouse ribosome profiling samples, 5, 5, 5, 3, 3, and 4 biological replicates were used for GV, MII, 1-cell, 2-cell, 4-cell and 8-cell stages, respectively. In mouse RNA-Seq samples, 4, 4, 4, 4, 2 and 4 biological replicates were used in GV, MII, 1-cell, 2-cell, 4-cell and 8-cell stages. For experiments with K562 cells, we used 5 single cells, and 3 biological replicates each for the 100-cell and 10M-cell samples.
RNA recovery quantifications in Extended Data Fig. 1f were from four independent experiments. Size selection stringency (Fig. 2) was calculated from three experiments on two independent days.

Randomization

No randomization was performed. This is an observational study and there was no active manipulation of an independent variable.

Blinding

Data collection and analyses were carried out by different researchers. Blinding was not relevant due to the observational nature of the study.

Reporting for specific materials, systems and methods

We require information from authors about some types of materials, experimental systems and methods used in many studies. Here, indicate whether each material, system or method listed is relevant to your study. If you are not sure if a list item applies to your research, read the appropriate section before selecting a response.

Materials & experimental systems

n/a	Involved in the study	
☒	☐	Antibodies
☐	☒	Eukaryotic cell lines
☒	☐	Palaeontology and archaeology
☐	☒	Animals and other organisms
☒	☐	Human research participants
☒	☐	Clinical data
☒	☐	Dual use research of concern

Methods

n/a	Involved in the study	
☒	☐	ChIP-seq
☒	☐	Flow cytometry
☒	☐	MRI-based neuroimaging

Eukaryotic cell lines

Policy information about cell lines

Cell line source(s)

We used K562 human cell lines.

Authentication

The identity of K562 cell line was validated using STR profiling from ATCC.

Mycoplasma contamination

Periodically tested for lack of mycoplasma contamination (at least every six months).

Commonly misidentified lines (See ICLAC register)	There are no misidentified lines.

Animals and other organisms

Policy information about studies involving animals; ARRIVE guidelines recommended for reporting animal research

Laboratory animals	We used the two mouse strains: C57BL/6J (female) and CAST/EiJ (male). Mice were ~8 weeks old at the time of the experiment.
Wild animals	There are no wild animals involved in this study.
Field-collected samples	There are no field-collected samples in this study.
Ethics oversight	All experiments using mice were done at the Mouse Genetic Engineering Facility and were approved by the Institutional Animal Care and Use Committee at the University of Texas at Austin.

Note that full information on the approval of the study protocol must also be provided in the manuscript.
